# Machine Learning-Based Multiclass Classification of Hb E Carrier Status and Thalassemia-Related Hematological Profiles Using Routine Complete Blood Count Parameters

**DOI:** 10.3390/bioengineering13101118

**Published:** 2026-09-24

**Authors:** Mert Basancelebi, Irem Senyer Yapici, Rukiye Uzun Arslan

**Affiliations:** 1 Department of Electrical and Electronics Engineering, Zonguldak Bulent Ecevit University, 67100 Zonguldak, Türkiye; mert.basancelebi@beun.edu.tr; 2 Department of Computer Engineering, Zonguldak Bulent Ecevit University, 67100 Zonguldak, Türkiye; senyerirem@beun.edu.tr

**Keywords:** complete blood count, Hb E carrier status, hereditary hemoglobin disorders, machine learning, class imbalance, thalassemia, CatBoost

## Abstract

Routine complete blood count (CBC) measurements are widely available and may support the initial assessment of thalassemia-related hematological conditions. However, overlapping hematological profiles and class imbalance can complicate differentiation among groups. This study evaluated seven supervised machine learning classifiers for multiclass classification of normal hematological profiles, suspected α-thalassemia profiles, Hb E carrier-related profiles, and other thalassemia-related/hemoglobinopathic profiles using a publicly available CBC dataset. An unresampled baseline was compared with four synthetic oversampling strategies: SMOTE, Borderline-SMOTE, SVM-SMOTE, and KMeans-SMOTE. Model development and hyperparameter optimization were performed within five-fold stratified cross-validation, with model selection based on cross-validated Matthews correlation coefficient (MCC). The untouched internal hold-out test set was reserved for final evaluation. CatBoost without resampling yielded the highest cross-validated MCC (0.6378). On the internal hold-out test set, it achieved an accuracy of 0.7950, an MCC of 0.6537, Cohen’s kappa of 0.6499, and micro- and macro-average ROC–AUC values of 0.9554 and 0.9227, respectively. However, class-specific sensitivity was heterogeneous, ranging from 0.4028 for suspected α-thalassemia to 0.9767 for the normal hematological profile. Synthetic oversampling did not consistently increase overall CV MCC, although sensitivity improved for some minority classes. These findings characterize the performance and limitations of CBC-based multiclass classification and indicate the need for further external and clinically oriented validation before screening or referral applications can be considered.

## 1. Introduction

Inherited hematological diseases, including thalassemia syndromes and structural hemoglobin variants, constitute an important global health burden because of their high prevalence, genetic transmission, and heterogeneous clinical manifestations [1,2,3]. Accurate and timely identification of these disorders is essential for determining the need for further hematological investigation, molecular confirmation, genetic counseling, and appropriate clinical follow-up. Although advances in molecular diagnostics and precision medicine have improved the characterization of hereditary hemoglobin disorders, access to specialized tests may remain limited in resource-constrained settings. Routine complete blood count (CBC) parameters therefore represent accessible and clinically relevant hematological biomarkers that may support preliminary screening and referral decisions. However, substantial overlap among the CBC profiles of different thalassemia types, hemoglobin variants, and hematologically normal individuals limits the discriminatory capacity of single indices and fixed threshold-based decision rules. This screening uncertainty is particularly relevant in mild, carrier, or atypical cases in which further testing may not initially be considered.

Thalassemia syndromes result from reduced or absent synthesis of one or more globin chains, whereas structural hemoglobin variants, such as hemoglobin E (Hb E), arise from qualitative alterations in the hemoglobin molecule [2,4]. Despite their different molecular mechanisms, these conditions may produce similar changes in erythrocyte-related CBC parameters, including mean corpuscular volume, mean corpuscular hemoglobin, red blood cell count, and hemoglobin concentration. Hb E carrier status represents a particularly challenging screening problem because carriers may exhibit only mild microcytosis or near-normal hematological findings. Consequently, their CBC profiles may overlap with those of normal individuals or patients with other thalassemia-related patterns. Nevertheless, identifying Hb E carrier status remains clinically relevant because its co-inheritance with α- or β-thalassemia may result in more complex and, in some cases, more severe hematological phenotypes. A screening approach capable of recognizing multivariable relationships among routine hematological parameters may therefore capture information that is not reflected by individual CBC indices alone.

To address the limitations of rule-based screening, statistical and machine learning (ML) methods have increasingly been investigated for the classification of hemoglobinopathies and thalassemia-related conditions using routinely collected hematological data [5,6,7,8,9,10,11,12,13,14,15,16]. Previous studies have evaluated algorithms such as support vector machines (SVM), decision trees (DTs), neural networks, and ensemble learning models and have generally reported that ML-based methods can outperform conventional discriminant indices in selected classification tasks [8,10,14,17,18,19,20,21,22,23]. Schipper et al. [10], for example, reported that an Extreme Gradient Boosting (XGBoost) model based on routine CBC parameters achieved area under the receiver operating characteristic curve values ranging from 0.94 to 0.97 for the prediction of hemoglobinopathies, including structural variants and Hb E. Saha et al. [24] found that XGBoost and Skope-Rules provided strong multiclass discrimination among β-thalassemia carriers, individuals with iron-deficiency anemia, and Hb E carriers in a heterogeneous population. Christensen et al. [13] reported that deep learning and conventional ML models were more effective than traditional hematological indices for identifying α-thalassemia-related profiles. Similarly, Paydaş Hataysal and Körez [23] showed that models based on routine erythrocyte indices could distinguish β-thalassemia carriers, individuals with iron-deficiency anemia, and healthy individuals with high predictive performance. Almasallati et al. [25] also demonstrated the utility of clustering-based ML for classifying multiple anemia subtypes from CBC data. Collectively, these studies suggest that CBC-derived parameters may provide informative inputs for machine-learning-based hematological classification, although the reported performance is not directly comparable across studies because of differences in target definitions, study populations, and validation designs.

Despite these advances, several methodological limitations remain. Existing studies frequently evaluate a restricted range of learning algorithms, focus on binary or narrowly defined classification problems, or report performance using a limited set of evaluation metrics [8,13,14,20,21,26,27,28]. Class imbalance is another important concern because clinically relevant hematological groups, including carrier classes, may be underrepresented relative to normal or more prevalent categories. In such cases, a model may achieve acceptable overall accuracy while showing limited sensitivity for less frequent classes. Resampling methods such as the Synthetic Minority Over-Sampling Technique (SMOTE), Adaptive Synthetic Sampling, SMOTE–Tomek Links, and SMOTE combined with Edited Nearest Neighbors have been used to address this issue [21,29]. However, the effect of oversampling may vary depending on the data distribution and the learning algorithm, and its benefit should therefore be assessed against an unresampled baseline using a leakage-free validation procedure. Recent biomedical ML studies have similarly shown that the effect of class-imbalance handling is context-dependent and should be evaluated within an appropriate validation framework rather than assumed to improve predictive performance a priori [30,31]. This consideration is particularly relevant to Hb E carrier identification, where class imbalance and overlap with other CBC profiles may affect classification performance.

Our previous study using the same publicly available CBC dataset focused on ensemble-model performance and SHAP-based interpretation for the same four-class hematological classification task [32]. The present study addresses a distinct methodological question and extends the analysis beyond a change in model emphasis. Specifically, it examines whether interventions intended to address class imbalance produce consistent improvements across learning algorithms and performance dimensions, or instead introduce trade-offs between minority-class detection and overall multiclass performance.

The substantive contribution of the present study therefore lies in a controlled assessment of class-imbalance handling rather than in the proposal of a new classifier or resampling algorithm. An unresampled baseline and four synthetic oversampling strategies were evaluated across seven classifiers within the same leakage-free validation framework. Importantly, the comparison was extended beyond aggregate accuracy and ROC–AUC to include class-specific sensitivity, precision–recall behavior, balanced accuracy, calibration, and threshold-dependent false-negative trade-offs. Repeated cross-validation, corrected paired statistical comparisons, and uncertainty estimates were additionally used to determine whether apparent differences between configurations were stable rather than attributable to sampling variability.

Accordingly, the study was designed to determine (i) whether the effect of synthetic oversampling is consistent across learning algorithms; (ii) whether improvements in minority-class sensitivity are accompanied by changes in precision, specificity, or overall classification agreement; and (iii) whether the observed differences remain stable when performance variability and statistical uncertainty are considered. This framework provides a more detailed characterization of the consequences of class-imbalance handling in multiclass CBC-based hematological classification than model ranking alone.

## 2. Materials and Methods

This section describes the methodological framework used in the present study. The overall workflow is illustrated in Figure 1. The dataset was first divided into stratified training and hold-out test subsets. Within the training set, preprocessing and resampling were incorporated into five-fold stratified cross-validation together with model fitting and hyperparameter optimization. An unresampled baseline and four synthetic oversampling strategies were compared, and model selection was based on cross-validated MCC. The hold-out test set remained untouched until final performance evaluation.

### 2.1. Dataset

In this study, a dataset publicly available through the Mendeley Data platform was used [33]. The dataset is based on CBC measurements obtained using routine automated hematology analyzers. It comprises 5778 individuals distributed across four hematological categories: 3224 in Class 0, 720 in Class 1, 1362 in Class 2, and 472 in Class 3. The overall class distribution is shown in Figure 2. The mean age of the study population was 32.35 ± 10.91 years, with a median age of 30 years (interquartile range: 26–36 years) and an overall range of 18–96 years. The mean ages were 31.29 ± 8.75 years for Class 0, 35.43 ± 14.12 years for Class 1, 31.50 ± 10.28 years for Class 2, and 37.38 ± 16.64 years for Class 3. Sex was provided in the public dataset as a binary-coded variable, with 3100 records coded as 0 and 2678 as 1. Because the correspondence between these numerical codes and the biological-sex categories could not be verified from the publicly available documentation, the codes were not relabeled in the descriptive analysis.

Class 0 represents individuals with a normal Hb fraction and normal CBC findings. Class 1 includes individuals with a normal Hb fraction but CBC parameters showing a possible α-thalassemia pattern. Class 2 includes individuals reported to have Hb E (Hb E carrier ± α-thalassemia) and represents a clinically relevant category. Class 3 corresponds to thalassemic or other hemoglobinopathic patterns other than Hb E. The original class definitions provided in the dataset were retained, and no reclassification was performed. The original class labels provided in the source dataset were retained without reclassification. According to the source study, hemoglobin-typing results were interpreted in accordance with the national laboratory diagnostic guidance of the Department of Medical Sciences, Thailand, and were used as a reference for class assignment [34]. However, the original labeling procedure was not entirely independent of the CBC findings. Both Class 0 and Class 1 were associated with a normal hemoglobin-typing pattern, while their operational distinction also incorporated CBC characteristics: Class 0 represented individuals with normal CBC findings, whereas Class 1 represented individuals with a CBC pattern considered compatible with possible α-thalassemia. Thus, CBC information contributed to the distinction between at least these two categories, which should be taken into account when interpreting the corresponding classification performance. The diagnostic workflow described by the source authors included hemoglobin typing by high-performance liquid chromatography (HPLC) or capillary electrophoresis; however, the public dataset does not specify the analytical method or instrument used for each individual record. Class 1 therefore represents a normal hemoglobin-typing pattern with possible α-thalassemia rather than molecularly confirmed α-thalassemia, as definitive α-thalassemia status requires molecular testing [34]. Class 2 similarly includes Hb E trait with or without co-inherited α-thalassemia. Accordingly, the four labels are treated in the present study as dataset-defined hematological categories rather than independently adjudicated or molecularly confirmed clinical diagnoses.

The source dataset contains routine hematological measurements together with demographic variables. For the primary comparative analysis, biological sex was excluded from the predictor matrix because the four numerical SMOTE variants evaluated in the main framework generate synthetic observations by interpolation in the feature space and do not explicitly preserve categorical-variable structure. Age was retained as a continuous covariate. The primary predictor set therefore comprised 20 variables: 19 routine CBC-derived parameters and age. Accordingly, the primary model should be regarded as a deliberately restricted predictor configuration designed to permit a consistent comparison of the four numerical oversampling strategies. The exclusion of sex was therefore a methodological design choice rather than an assumption that sex has no potential clinical or predictive relevance. Biological sex is associated with differences in several routine hematological measures, particularly red blood cell count, hemoglobin, and hematocrit, and sex-specific reference intervals have been reported in adult populations [35]. Therefore, sex could plausibly contribute predictive information through differences in baseline CBC profiles. However, the present dataset does not establish a direct clinical association between sex and the dataset-defined hematological categories; its contribution was therefore examined empirically in the sensitivity analyses rather than assumed a priori. The potential effect of including sex was examined separately in sensitivity analyses using categorical-compatible and non-resampling approaches. Table 1 summarizes the retained predictors and their clinical relevance. The univariate distributions of the variables included in the final analysis are presented in Supplementary Figure S1 for descriptive assessment.

### 2.2. Data Preprocessing and Class Imbalance Handling

The dataset was examined for missing values before model development, and no missing observations were identified; therefore, no imputation or observation removal was required. The original class labels provided in the dataset were retained without reclassification. Following exclusion of Sex from the predictor matrix, 20 numerical predictors were retained for the final analysis. The dataset was subsequently divided into stratified training and hold-out test subsets before any scaling or resampling was performed.

To minimize information leakage, preprocessing and resampling were restricted to the training data and were performed within the cross-validation pipeline. StandardScaler was fitted separately on the training portion of each fold. Class imbalance was addressed by comparing an unresampled baseline with four synthetic oversampling strategies: SMOTE, Borderline-SMOTE, SVM-SMOTE, and KMeans-SMOTE. For the oversampled conditions, the sampler was applied after standardization and before classifier fitting within each training fold. The corresponding validation fold and the hold-out test set were not used to fit either the scaler or the sampler. The hold-out test set retained its original class distribution and remained excluded from preprocessing, resampling, hyperparameter tuning, and model selection.

#### 2.2.1. No Resampling Baseline

An unresampled baseline was included to determine whether synthetic over-sampling improved performance relative to training on the original class distribution. In this condition, StandardScaler was applied within each training fold and the sampler step was passed through without modifying the class distribution. Including this baseline allows the effect of resampling to be interpreted directly rather than assuming that class balancing is necessarily beneficial.

#### 2.2.2. SMOTE

SMOTE is an oversampling method developed to address class imbalance and is based on synthetic sample generation. In this method, new samples are generated through linear interpolation between neighboring observations from the minority class, taking into account their local neighborhood structure. Unlike approaches based solely on replicating existing observations, SMOTE introduces additional synthetic samples into the feature space and may therefore provide greater variation within underrepresented classes [36,37]. In the present study, SMOTE was applied only within the training folds of the cross-validation procedure, after standardization and before classifier fitting. Consequently, no synthetic samples were introduced into the validation folds or the hold-out test set.

#### 2.2.3. Borderline-SMOTE

Borderline-SMOTE is an extension of SMOTE that focuses synthetic sample generation on minority-class observations located close to class boundaries, where the risk of misclassification is relatively high. In this approach, minority samples situated in difficult boundary regions are identified, and synthetic observations are generated primarily around these samples. Unlike standard SMOTE, which considers the minority-class distribution more broadly, Borderline-SMOTE places greater emphasis on regions in which the distinction between classes is less clear. This strategy is intended to improve the representation of difficult minority-class examples during model training [38,39]. In the present study, Borderline-SMOTE was applied only within the training folds of the cross-validation procedure, after standardization and before classifier fitting.

#### 2.2.4. SVM-SMOTE

SVM-SMOTE is a SMOTE-based oversampling method that uses a support vector machine to identify minority-class observations located near the decision boundary between classes. Synthetic samples are then generated primarily around these boundary regions, where the risk of misclassification may be higher. SVM-SMOTE extends boundary-focused synthetic oversampling by using support-vector information to guide sample generation near difficult class regions [40]. In the present study, SVM-SMOTE was applied only within the training folds of the cross-validation procedure, after standardization and before classifier fitting.

#### 2.2.5. KMeans-SMOTE

KMeans-SMOTE is a clustering-based oversampling method that generates synthetic samples by considering the local structure of minority-class observations in the feature space. In this method, the data are first partitioned into clusters using the K-means algorithm, after which synthetic observations are generated within selected minority-dominated regions using SMOTE. By distributing synthetic sampling across different local regions, KMeans-SMOTE is intended to better represent heterogeneous minority-class patterns and reduce the tendency to concentrate synthetic samples in a limited portion of the feature space [41]. In the present study, KMeans-SMOTE was applied only within the training folds of the cross-validation procedure, after standardization and before classifier fitting.

### 2.3. Experimental Setup

#### 2.3.1. Data Splitting and Validation

To obtain a hold-out evaluation of model performance, all 5778 records were divided into training and hold-out test subsets after Sex had been excluded from the predictor matrix, using a stratified 80/20 split with a random state of 42. The training subset contained 4622 observations (Class 0: 2579; Class 1: 576; Class 2: 1089; Class 3: 378), whereas the hold-out test subset contained 1156 observations (Class 0: 645; Class 1: 144; Class 2: 273; Class 3: 94). Five-fold stratified cross-validation with shuffling and random_state = 42 was performed only on the training data. Within each fold, StandardScaler was fitted using the fold-specific training portion, followed by either no resampling or one of the four oversampling strategies and subsequent classifier fitting. The corresponding validation fold was not used to fit either the scaler or the sampler. The hold-out test set remained untouched throughout preprocessing, resampling, hyperparameter tuning, and model selection and was used only for final performance evaluation.

#### 2.3.2. Hyperparameter Optimization

Hyperparameter tuning was performed to identify suitable model configurations within predefined search spaces. GridSearchCV was applied only to the training data using five-fold stratified cross-validation. For each classifier and resampling strategy, preprocessing, resampling, and model fitting were incorporated into the same pipeline so that these operations were repeated independently within each cross-validation fold. The predefined hyperparameter combinations listed in Table 2 were evaluated, and Matthews correlation coefficient (MCC) was used as the refit criterion for selecting the best configuration. The hold-out test set was not used during hyperparameter tuning, classifier selection, or resampling-strategy selection and was reserved exclusively for final performance evaluation. Table 2 summarizes the hyperparameter search spaces considered for each classifier.

Based on the predefined training-set cross-validated MCC criterion, the final selected configuration was CatBoost without resampling. The selected CatBoost hyperparameters were 400 iterations, a learning rate of 0.05, a tree depth of 6, and an L2 leaf regularization coefficient of 3. The model used the MultiClass loss function, and a fixed random seed of 42 was used for reproducible stochastic procedures. These settings were retained for the final internal hold-out evaluation. All analyses were implemented in Python 3.14.1. Model development and cross-validation were performed using scikit-learn 1.8.0 [42], while the resampling procedures and leakage-controlled pipelines were implemented using imbalanced-learn 0.14.1 [43]. XGBoost 3.1.2 and CatBoost 1.2.10 were used for the corresponding boosting classifiers.

#### 2.3.3. Machine Learning Models

Seven supervised ML algorithms representing different learning strategies were evaluated: K-nearest neighbors (KNN), decision tree (DT), support vector classifier (SVC), Bagging, gradient boosting (GB), XGBoost, and CatBoost. These algorithms were selected to provide methodological diversity across instance-based, tree-based, kernel-based, and ensemble learning approaches. KNN is an instance-based classifier that assigns a class label according to the classes of the *k* nearest training observations in the feature space. As a non-parametric and distance-based method, its predictions are determined directly from the local structure of the training data [44,45]. DT is a tree-based method that recursively partitions the feature space according to predictor values. Its hierarchical structure provides relatively interpretable decision rules, although overly complex trees may be prone to overfitting [46,47]. SVC is a kernel-based classifier that seeks a maximum-margin decision boundary between classes and can model nonlinear class separation through the use of kernel functions [48]. Bagging is an ensemble-learning method that combines predictions from multiple base learners trained on bootstrap samples of the training data, primarily with the aim of reducing model variance and improving prediction stability [49,50]. GB is an ensemble method in which weak learners are trained sequentially, with each subsequent learner focusing on errors remaining from the preceding stages [51,52]. XGBoost extends the gradient-boosting framework through regularization and computationally efficient optimization, providing additional control over model complexity [53,54]. CatBoost is another GB algorithm that uses ordered boosting and symmetric tree structures to reduce prediction shift and support model training [55,56].

### 2.4. Performance Metrics

Model performance was evaluated using complementary metrics reflecting overall classification performance, agreement between predicted and true labels, and class-specific error behavior. The overall evaluation measures included accuracy, balanced accuracy, macro-averaged specificity, macro-averaged false positive rate (FPR), Matthews correlation coefficient (MCC), and Cohen’s kappa. In addition, class-specific sensitivity (recall), specificity, FPR, precision, F1-score, and one-vs-rest receiver operating characteristic area under the curve (ROC–AUC) were calculated on the hold-out test set. For multiclass specificity and FPR, each class was treated as the positive class against all remaining classes, and the resulting class-specific values were macro-averaged. ROC–AUC was calculated from class-probability estimates using a one-vs-rest formulation, with both micro- and macro-average values reported as summary measures. Precision–recall (PR) curves were also generated for each class using a one-vs-rest formulation, and class-specific average precision was reported as a summary of PR performance. These measures were included to provide additional information on performance in the less frequent classes. Cross-validated MCC was used as the predefined criterion for hyperparameter optimization and model and resampling-strategy selection, whereas the remaining metrics were used to provide complementary characterization of overall and class-specific performance.

MCC was selected as the primary optimization criterion because it provides a single overall measure of agreement that incorporates the full multiclass confusion structure and is less dominated by the majority class than accuracy in an imbalanced setting. This choice was intended to provide a consistent criterion for the methodological comparison of classifier–strategy combinations rather than to define the clinically preferred operating objective for a screening application. In a screening context, class-specific sensitivity and false-negative rates may warrant greater emphasis, depending on the intended population and clinical consequences of missed cases.

For a multiclass problem with *K* classes, let *C* denote the confusion matrix, where *C_ij_* represents the number of observations belonging to class *i* that were predicted as class *j*. Accuracy represents the proportion of all observations that are correctly classified and is defined as:

(1) $$\mathrm{Accuracy}=\frac{\sum_{k=1}^{K}C_{\mathrm{kk}}}{N}$$

where *N* denotes the total number of observations. For each class *k*, true positives (*TP_k_*), true negatives (*TN_k_*), false positives (*FP_k_*), and false negatives (*FN_k_*) were determined using a one-vs-rest formulation. Class-specific specificity, which represents the proportion of observations not belonging to class *k* that are correctly identified as negative, was calculated as:

(2) $${\mathrm{Specificity}}_{k}=\frac{TN_{k}}{TN_{k}+FP_{k}}$$

The overall specificity was summarized using the unweighted macro-average across all classes:

(3) $${\mathrm{Specificity}}_{\mathrm{macro}}=\frac{1}{K}\sum_{k=1}^{K}{\mathrm{Specificity}}_{k}$$

The class-specific FPR represents the proportion of negative observations incorrectly assigned to class *k* and was calculated as:

(4) $${\mathrm{FPR}}_{k}=\frac{FP_{k}}{FP_{k}+TN_{k}}=1-{\mathrm{Specificity}}_{k}$$

The macro-averaged FPR was obtained as:

(5) $${\mathrm{FPR}}_{\mathrm{macro}}=\frac{1}{K}\sum_{k=1}^{K}{\mathrm{FPR}}_{k}$$

Sensitivity, also referred to as recall, represents the proportion of observations belonging to class *k* that are correctly identified:

(6) $${\mathrm{Sensitivity}}_{k}=\frac{TP_{k}}{TP_{k}+FN_{k}}$$

Precision represents the proportion of observations predicted as class *k* that actually belong to that class:

(7) $${\mathrm{Precision}}_{k}=\frac{TP_{k}}{TP_{k}+FP_{k}}$$

The F1-score provides a harmonic-mean summary of precision and sensitivity and was calculated as:

(8) $$F1_{k}=2x\frac{{\mathrm{Precision}}_{k}\times {\mathrm{Sensitivity}}_{k}}{{\mathrm{Precision}}_{k}+{\mathrm{Sensitivity}}_{k}}$$

Because the present study involves an imbalanced multiclass classification problem, MCC was used as a complementary overall performance measure [57]. For the multiclass case, MCC was calculated as:

(9) $$\mathrm{MCC}=\frac{\mathrm{cs}-\sum_{k=1}^{K}p_{k}t_{k}}{\sqrt{(s^{2}-\sum_{k=1}^{K}p_{k}^{2})(s^{2}-\sum_{k=1}^{K}t_{k}^{2})}}$$

where $c=\sum_{k=1}^{K}C_{\mathrm{kk}}$ is the total number of correctly classified observations, *s* = *N* is the total number of observations, *p_k_* is the number of observations predicted as class *k*, and *t_k_* is the number of observations whose true label is class *k*. MCC ranges from −1 to 1, with values closer to 1, indicating stronger agreement between predicted and true class assignments.

Cohen’s kappa (κ) was used to quantify agreement between predicted and true labels while accounting for agreement expected by chance [58]. Its mathematical formulation is expressed as follows:

(10) $$\kappa =\frac{p_{o}-p_{e}}{1-p_{e}}$$

where *p_o_* denotes the observed proportion of agreement and *p_e_* denotes the expected agreement by chance.

ROC–AUC was calculated using one-vs-rest class probabilities [59]. A separate AUC value was obtained for each class by treating that class as positive and all remaining classes as negative. The micro-average AUC summarizes discrimination after pooling the one-vs-rest decisions across all classes, whereas the macro-average AUC represents the unweighted mean of the class-specific AUC values.

Balanced accuracy was calculated as the unweighted mean of class-specific recall values and was included to reduce the influence of the majority class on the overall assessment of model performance. Probability calibration was additionally assessed on the untouched hold-out set using the multiclass Brier score, macro-averaged one-vs-rest Brier score, and class-specific expected calibration error (ECE). Calibration curves were generated for each class using one-vs-rest predicted probabilities and are provided in Supplementary Figure S2. These analyses were used to characterize the agreement between predicted probabilities and observed class frequencies; no post hoc recalibration procedure was fitted to the hold-out data. To further examine class-specific operating characteristics, threshold-dependent analyses were performed using one-vs-rest class probabilities. For the less frequent classes, sensitivity, specificity, precision, negative predictive value, false-positive rate, and false-negative rate were evaluated across a range of candidate probability thresholds. These analyses were intended to characterize the trade-off between case detection and classification errors rather than to identify a clinically validated operating threshold. Because no clinically acceptable false-negative rate or decision threshold was prespecified in the source dataset, the threshold analyses were considered exploratory and no probability threshold was proposed for clinical use. Detailed threshold-dependent operating characteristics for Classes 1–3 are provided in Supplementary Table S3.

### 2.5. Sensitivity Analyses

Additional sensitivity analyses were conducted to examine whether the exclusion of biological sex from the primary predictor set materially affected model performance. The primary 20-predictor configuration, consisting of 19 CBC-derived variables and age, was compared with an expanded predictor set that also included sex. Because sex is a categorical variable, analyses incorporating this predictor used categorical-compatible handling rather than ordinary numerical SMOTE interpolation. SMOTENC was additionally evaluated as a resampling approach that distinguishes categorical and continuous predictors. A class-weighted CatBoost configuration was also examined as a non-resampling alternative for addressing class imbalance. These analyses were treated as sensitivity analyses and were not used to redefine the primary model-selection framework.

### 2.6. Stability and Statistical Comparison of Model Performance

To assess the stability of the observed differences beyond a single five-fold cross-validation split, all 35 tuned classifier–strategy configurations were further evaluated using repeated stratified cross-validation with five folds and ten repetitions. Performance distributions were summarized using the mean, standard deviation, and corrected 95% confidence intervals. The same resampling splits were used for all configurations. The configuration selected by the original training-set CV MCC criterion was compared with each alternative using paired fold-level MCC differences and a corrected resampled *t*-test to account for dependence among repeated cross-validation estimates. Holm-adjusted *p*-values were used to account for multiple comparisons. In addition, bootstrap resampling of the untouched hold-out predictions was used to estimate 95% confidence intervals for the main performance measures.

### 2.7. Model Interpretability Methodas

Post hoc interpretability analyses were performed for the final selected model to examine the relative contribution of the input variables to model predictions. Model-agnostic permutation importance was calculated on the untouched internal hold-out set by quantifying the reduction in MCC after randomly permuting each predictor while leaving the remaining variables unchanged. For the selected CatBoost model, SHAP values were additionally calculated to characterize feature contributions at both the overall and class-specific levels. Global importance was summarized using the mean absolute SHAP value for each predictor, while class-specific SHAP values were used to examine whether the relative contribution of individual variables differed across the four hematological categories. These analyses were performed after model selection and were not used for hyperparameter tuning or selection of the final model. Global SHAP feature importance and model-agnostic permutation importance are presented in Supplementary Figures S3 and S4, respectively, while the highest class-specific SHAP contributions are summarized in Supplementary Table S4.

## 3. Results

This study evaluated the multiclass classification of hematological profiles using seven supervised ML algorithms under different class-imbalance handling strategies. The four dataset-defined classes included normal hematological profiles, suspected α-thalassemia profiles, Hb E carrier-related profiles, and other thalassemia-related/hemoglobinopathic profiles. To examine the effect of class balancing on model performance, an unresampled baseline was compared with four synthetic oversampling strategies: SMOTE, Borderline-SMOTE, SVM-SMOTE, and KMeans-SMOTE. The original dataset was first divided into stratified training and hold-out test subsets using an 80/20 split. All preprocessing, oversampling, model fitting, and hyperparameter optimization procedures were restricted to the training data, with preprocessing and resampling performed within five-fold stratified cross-validation. Model selection was based on cross-validated MCC, whereas the untouched internal hold-out test set was used only for final performance evaluation. A total of 35 classifier–strategy combinations were evaluated. The cross-validation and internal hold-out test results are presented in the following subsections.

### 3.1. Cross-Validated Performance Across Classifiers and Class-Imbalance Strategies

The performance of the seven classifiers was first compared across the unresampled baseline and the four synthetic oversampling strategies. Because model selection was based on cross-validated MCC, the comparison focused on CV MCC across the classifier–strategy combinations. The corresponding results are presented in Table 3.

CatBoost yielded the highest CV MCC within each of the five evaluated strategies, although XGBoost and GB produced relatively close values in several conditions. CatBoost CV MCC ranged from 0.6122 with Borderline-SMOTE to 0.6378 without resampling. The highest CV MCC among the 35 evaluated combinations was observed for CatBoost without resampling (0.6378). The corresponding values for CatBoost with KMeans-SMOTE and SVM-SMOTE were 0.6311 and 0.6293, respectively.

When the results were considered at the strategy level, the unresampled condition had the highest mean CV MCC across the seven classifiers (0.6183). This was followed by KMeans-SMOTE (0.6069), SVM-SMOTE (0.5991), SMOTE (0.5797), and Borderline-SMOTE (0.5760). For each of the seven classifiers, the highest CV MCC was observed under the unresampled condition. Thus, within the present dataset and evaluation framework, synthetic oversampling did not lead to a consistent increase in CV MCC relative to training without resampling.

Based on the predefined CV MCC criterion, CatBoost was selected within each class-imbalance strategy, and CatBoost without resampling was retained as the final configuration. The internal hold-out test performance of these five strategy-specific CatBoost models is summarized in Table 4.

On the untouched internal hold-out test set, the selected unresampled CatBoost model achieved an accuracy of 0.7950, a macro-averaged specificity of 0.9196, a macro-averaged FPR of 0.0804, an MCC of 0.6537, and Cohen’s kappa of 0.6499. Its micro- and macro-average ROC–AUC values were 0.9554 and 0.9227, respectively. The hold-out results of the five CatBoost configurations were relatively close for several metrics. For example, SVM-SMOTE yielded a slightly higher macro-averaged specificity (0.9224) and a slightly lower FPR (0.0776) than the unresampled configuration. These hold-out differences did not affect model selection, which had been determined beforehand using CV MCC.

### 3.2. Class-Specific Performance Across Class-Imbalance Strategies

To examine whether the class-imbalance strategy was associated with differences in class-level performance, the class-specific internal hold-out test results of the CatBoost models selected within each strategy were compared. Sensitivity, specificity, FPR, precision, F1-score, and one-vs-rest AUC were considered for each of the four classes. The corresponding results are presented in Table 5.

Table 5 shows that the class-specific results varied across the evaluated resampling strategies. For Class 0, sensitivity remained above 0.93 under all five conditions and was highest without resampling (0.9767). The corresponding one-vs-rest AUC values were also similar across strategies, ranging from 0.9737 to 0.9752. The unresampled model had a lower Class 0 specificity (0.8513) than the oversampled configurations, corresponding to a higher false-positive rate for this class.

For Class 1, sensitivity ranged from 0.4028 without resampling to 0.5556 with SMOTE. SMOTE also yielded the highest F1-score for this class (0.5674), compared with 0.4957 without resampling. In contrast, the unresampled configuration yielded the highest precision (0.6444), specificity (0.9684), and AUC (0.9297) among the five evaluated strategies.

For the Hb E carrier-related Class 2, sensitivity was 0.6740 without resampling and increased to 0.6960 with both SVM-SMOTE and KMeans-SMOTE. The highest F1-score for this class was observed with SVM-SMOTE (0.6597), although the value obtained without resampling was similar (0.6560). The class-specific AUC ranged from 0.8735 to 0.8839, with the highest value observed for the unresampled configuration.

For Class 3, sensitivity ranged from 0.4894 to 0.5532. SMOTE and Borderline-SMOTE produced the highest sensitivity (0.5532), whereas KMeans-SMOTE yielded the highest precision (0.6667) and specificity (0.9783). The highest F1-score and AUC for Class 3 were observed without resampling, with values of 0.5663 and 0.9018, respectively.

Overall, no single oversampling strategy yielded the highest values across all class-specific metrics. In some cases, oversampling was associated with higher sensitivity for the less frequent classes, whereas changes in precision, specificity, F1-score, and AUC varied according to the class and resampling strategy.

### 3.3. Detailed Evaluation of the Selected Model

The class-specific performance of the selected CatBoost model without resampling was further examined using the confusion matrix and one-vs-rest ROC curves. As shown in Figure 3, 630 of the 645 Class 0 observations were correctly classified, corresponding to a sensitivity of 0.9767. For Class 1, 58 of 144 observations were correctly identified, while 67 were classified as Class 2 and 19 as Class 3. Among the 273 Class 2 observations, 184 were correctly classified, with the most frequent misclassification occurring as Class 0 (*n* = 65). For Class 3, 47 of 94 observations were correctly identified; among the misclassified observations, 22 were assigned to Class 2, 14 to Class 1, and 11 to Class 0.

The class-specific performance measures showed differences among the four hematological categories. Consistent with the class-specific results in Table 5, the confusion matrix showed that correct classification was most frequent for Class 0, whereas a larger proportion of errors was observed for Classes 1–3.

The class-specific and micro-average one-vs-rest ROC curves for the selected CatBoost model without resampling are presented in Figure 4. The class-specific AUC values were 0.9752 for Class 0, 0.9297 for Class 1, 0.8839 for Class 2, and 0.9018 for Class 3. The micro-average ROC curve yielded an AUC of 0.9554, while the macro-average AUC calculated across the class-specific one-vs-rest results was 0.9227. Class 2 had the lowest one-vs-rest AUC among the four classes, whereas Class 1 showed the lowest sensitivity and F1-score. Thus, the relative difficulty of class discrimination varied according to the performance measure considered.

### 3.4. Complementary Performance, Uncertainty, and Calibration Analysis

In addition to the primary performance measures, the selected CatBoost model without resampling achieved a balanced accuracy of 0.6384 and a macro-F1 score of 0.6627 on the internal hold-out set. The micro- and macro-average precision–recall performance, summarized by average precision (AP), was 0.8876 and 0.7146, respectively. Class-specific AP values further demonstrated heterogeneous precision–recall performance across the four categories, with values of 0.9728 for Class 0, 0.6319 for Class 1, 0.7071 for Class 2, and 0.5467 for Class 3. The corresponding bootstrap 95% confidence intervals were 0.9573–0.9850, 0.5504–0.7101, 0.6515–0.7627, and 0.4456–0.6665, respectively. The lower AP values observed for Classes 1 and 3 relative to the majority Class 0 further indicate that overall performance should not be interpreted without considering class-specific precision–recall behavior. Corresponding one-vs-rest precision–recall curves are provided in Supplementary Figure S5.

Bootstrap analysis based on 2000 resamples yielded 95% confidence intervals of 0.6182–0.6907 for MCC, 0.6039–0.6752 for balanced accuracy, 0.6250–0.6994 for macro-F1, and 0.9095–0.9348 for macro-average ROC–AUC. The corresponding 95% confidence interval for macro-average AP was 0.6794–0.7559.

Class-specific uncertainty estimates further emphasized the heterogeneous performance across the four categories. In particular, sensitivity for Class 1 was 0.4028 (95% bootstrap CI: 0.3235–0.4830), corresponding to a false-negative rate of 0.5972 (95% CI: 0.5170–0.6765). Thus, the relatively high one-vs-rest ROC–AUC for Class 1 (0.9297) did not translate into comparably high sensitivity at the default multiclass decision rule.

Probability-based evaluation of the selected model yielded a multiclass log loss of 0.5058, a macro-averaged one-vs-rest Brier score of 0.0711, and a macro-averaged expected calibration error of 0.0226. These measures and the corresponding class-specific calibration curves (Supplementary Figure S2) were used to characterize internal probability calibration rather than to establish clinical calibration.

Exploratory one-vs-rest threshold analyses further illustrated the trade-off between case detection and other operating characteristics. For Class 1, a probability threshold of 0.20 yielded a sensitivity of 0.8889, specificity of 0.8202, PPV of 0.4129, and NPV of 0.9811. At a threshold of 0.40, sensitivity decreased to 0.4931, whereas specificity and PPV increased to 0.9585 and 0.6283, respectively, with an NPV of 0.9300. Similar threshold-dependent trade-offs were observed for Classes 2 and 3, as summarized in Supplementary Table S3. These analyses were treated as descriptive, and no clinically validated probability threshold was selected from the internal hold-out data.

### 3.5. Repeated Cross-Validation and Statistical Comparisons

To further assess the stability of the model comparisons, repeated stratified 5-fold cross-validation with 10 repetitions was performed using the hyperparameters selected during the initial tuning stage. Corrected paired comparisons of MCC were then conducted between the selected No Resampling–CatBoost configuration and the alternative configurations, with Holm adjustment for multiple comparisons. The selected configuration showed a mean MCC advantage of 0.0363 over SMOTE–CatBoost (Holm-adjusted *p* = 0.0026) and 0.0382 over Borderline-SMOTE–CatBoost (Holm-adjusted *p* = 0.0033). In contrast, the mean MCC differences relative to SVM-SMOTE–CatBoost (0.0177, Holm-adjusted *p* = 0.2673) and KMeans-SMOTE–CatBoost (0.0154, Holm-adjusted *p* = 0.2673) were not statistically significant after correction for multiple comparisons. Similarly, the differences between the selected CatBoost configuration and the strongest unresampled alternatives, GB (mean MCC difference = 0.0063, Holm-adjusted *p* = 0.5332) and XGBoost (mean MCC difference = 0.0062, Holm-adjusted *p* = 0.5332), were not statistically significant. These findings indicate that although the No Resampling–CatBoost configuration achieved the highest cross-validated MCC and was retained according to the predefined selection criterion, its advantage was not uniform across all alternative configurations. Full corrected pairwise comparisons between the selected configuration and all alternative model–resampling configurations are provided in Supplementary Table S1. Repeated cross-validation summary statistics, including mean MCC, standard deviation, and corrected 95% confidence intervals for all 35 classifier–strategy configurations, are provided in Supplementary Table S1A.

### 3.6. Sensitivity Analyses for Predictor Composition and Class-Imbalance Handling

Focused sensitivity analyses were conducted to examine whether the exclusion of Sex or the use of alternative class-imbalance handling approaches materially affected model performance. The primary CBC + Age configuration achieved a hold-out MCC of 0.6537, balanced accuracy of 0.6384, and macro-F1 score of 0.6627. Removing Age and using CBC variables alone resulted in similar overall performance (MCC = 0.6548, balanced accuracy = 0.6315, macro-F1 = 0.6571), while adding Sex to the CBC + Age predictor set did not improve overall performance (MCC = 0.6504, balanced accuracy = 0.6337, macro-F1 = 0.6611).

Alternative imbalance-handling approaches changed the balance between overall multiclass performance and minority-class sensitivity. Class-weighted CatBoost increased balanced accuracy to 0.6468 but reduced MCC to 0.6399. Similarly, the SMOTENC-based analysis including Sex increased Class 1 sensitivity from 0.4028 in the primary configuration to 0.5417 and yielded a balanced accuracy of 0.6497, but this was accompanied by a lower overall MCC (0.6286), accuracy (0.7742), and macro-F1 (0.6468). Class 2 sensitivity also decreased from 0.6740 in the primary configuration to 0.5824 with SMOTENC. These sensitivity analyses therefore did not indicate a consistent overall performance benefit from including Sex and showed that categorical-compatible resampling and class weighting primarily altered the trade-off between minority-class sensitivity and overall multiclass performance rather than producing uniform improvement. Detailed results are provided in Supplementary Table S2.

### 3.7. Model Interpretability Analysis

Post hoc interpretability analysis showed that the selected No Resampling–CatBoost model relied predominantly on erythrocyte-related CBC indices. Global SHAP analysis identified MCV as the most influential predictor (mean |SHAP| = 1.0907), followed by MCH (0.3751), RDW (0.1504), and MCHC (0.1087) (Supplementary Figure S3). HGB and RBC showed smaller global SHAP contributions of 0.0517 and 0.0500, respectively.

The model-agnostic permutation analysis showed a similar overall pattern. Permuting MCV produced the largest mean reduction in MCC (0.5777 ± 0.0238), followed by RDW (0.0418 ± 0.0077), MCH (0.0336 ± 0.0096), and MCHC (0.0223 ± 0.0051) (Supplementary Figure S4). In contrast, Age produced a near-zero change in MCC under permutation (−0.0006 ± 0.0038), suggesting limited measurable contribution to the fitted model under this assessment.

Class-specific SHAP analysis further showed that MCV had the highest mean absolute SHAP value in each of the four hematological categories. MCH, RDW, and MCHC also appeared consistently among the dominant class-specific predictors, although their relative contributions differed across categories. For example, MCV was particularly prominent for Class 0 and Class 1, whereas RDW had a contribution similar in magnitude to MCV for Class 3. The five highest class-specific SHAP values for each category are provided in Supplementary Table S4. These attribution results describe the behavior of the fitted model and should not be interpreted as evidence of biological causality.

## 4. Discussion

This study compared seven supervised ML algorithms under an unresampled baseline and four synthetic oversampling strategies for the multiclass classification of four hematological profiles. The main finding was that CatBoost yielded the highest cross-validated MCC within each of the five class-imbalance conditions, while the highest overall CV MCC was obtained without resampling (0.6378). At the strategy level, the unresampled condition also produced the highest mean CV MCC across the seven classifiers. However, the repeated-cross-validation analysis showed that the numerical advantage of the selected No Resampling–CatBoost configuration was not statistically significant relative to several strong alternatives after correction for multiple comparisons, including the unresampled GB and XGBoost configurations and the CatBoost configurations combined with SVM-SMOTE and KMeans-SMOTE. Thus, selection of the final configuration reflects the predefined MCC-based model-selection criterion rather than evidence of uniform statistical superiority over all competing approaches. On the untouched internal hold-out test set, the selected unresampled CatBoost model achieved an accuracy of 0.7950, an MCC of 0.6537, Cohen’s kappa of 0.6499, and micro- and macro-average ROC–AUC values of 0.9554 and 0.9227, respectively. The differences among the leading CatBoost configurations were relatively small, and the results do not suggest a consistent overall advantage from synthetic oversampling in the present dataset. Sensitivity analyses did not indicate a consistent overall performance benefit from adding Sex to the primary predictor set. However, the SMOTENC-based analysis showed that categorical-compatible inclusion of Sex altered class-specific sensitivity, indicating a trade-off rather than a uniform performance improvement.

The performance observed with tree-based boosting methods is broadly consistent with previous studies in which ensemble learning approaches have been applied to hematological classification using routine blood parameters. Schipper et al. [10], for example, reported favorable discrimination with XGBoost models developed from routine CBC measurements for the prediction of hemoglobinopathies. Saha et al. [24] also evaluated XGBoost and other ML approaches for distinguishing β-thalassemia carriers, iron-deficiency anemia, and Hb E carriers. Although the present study differs in class definitions, validation design, and model-selection procedure, these studies similarly suggest that nonlinear combinations of routine hematological parameters can contain information relevant to the differentiation of hemoglobin-related conditions. Direct numerical comparisons should nevertheless be made cautiously because of differences in study populations, outcome definitions, sample sizes, laboratory platforms, and validation procedures.

The interpretability findings provide additional context for the model predictions. Both global SHAP and permutation importance identified MCV as the dominant predictor, with MCH, RDW, and MCHC also contributing prominently. This pattern is broadly consistent with established hematological characteristics used in the assessment of thalassemia and related hemoglobin disorders. MCV and MCH reflect erythrocyte size and hemoglobin content, respectively, while RDW provides information on variation in red-cell size and MCHC reflects intracellular hemoglobin concentration. Changes in these erythrocyte-related indices are well recognized in thalassemia-related and hemoglobinopathic profiles and have also formed the basis of previous machine-learning approaches using routine red-cell parameters [2,4,18,20,23,24]. The predominance of these variables therefore appears hematologically plausible. Nevertheless, SHAP and permutation importance quantify how the fitted model uses the available predictors; they do not demonstrate that the identified variables have a causal relationship with the underlying hematological conditions.

An important observation in the present analysis was that synthetic oversampling did not consistently increase cross-validated MCC. For every classifier evaluated, the highest CV MCC was obtained under the unresampled condition, although the magnitude of the difference varied among algorithms. KMeans-SMOTE and SVM-SMOTE generally produced values closer to the unresampled baseline than SMOTE and Borderline-SMOTE, but none of the four oversampling approaches resulted in a higher CV MCC for the corresponding classifier. This finding does not imply that synthetic oversampling is inherently unsuitable for hematological data. Rather, its effect appears to depend on both the learning algorithm and the performance criterion being considered. Synthetic observations may alter local class boundaries and the relative representation of minority patterns, which can benefit some aspects of classification while affecting others. Previous work has similarly emphasized that the utility of oversampling depends on the structure and overlap of the underlying classes [29]. A similar pattern has been reported in a hematological prediction study, in which SMOTE was applied to an imbalanced acute myeloid leukemia dataset but did not improve the primary AUC-based performance measure [30]. This further supports evaluating synthetic oversampling empirically within each dataset and modeling framework rather than assuming a universal performance benefit.

The class-specific results illustrate this trade-off more clearly. For suspected α-thalassemia (Class 1), sensitivity increased from 0.4028 without resampling to 0.5556 with SMOTE, and the corresponding F1-score increased from 0.4957 to 0.5674. However, the unresampled model retained higher precision, specificity, and one-vs-rest AUC for this class. A similar pattern was observed for the Hb E carrier-related Class 2. Its sensitivity increased from 0.6740 without resampling to 0.6960 with SVM-SMOTE and KMeans-SMOTE, whereas the highest class-specific AUC was obtained without resampling (0.8839). These findings suggest that changes in class balance may shift the balance between sensitivity and other measures rather than improving all aspects of class-specific performance simultaneously.

These results are therefore better interpreted as a multi-objective trade-off rather than as evidence that oversampling is uniformly ineffective. The unresampled CatBoost configuration was retained because the primary model-selection criterion was predefined as cross-validated MCC, which summarizes overall multiclass classification performance. However, a screening-oriented application may place greater emphasis on sensitivity for clinically relevant carrier classes, even if this is accompanied by a modest reduction in overall MCC, precision, or specificity. The present retrospective analysis does not establish which trade-off would be clinically preferable, and such priorities would need to be defined prospectively according to the intended screening population and the consequences of false-negative and false-positive classifications.

Taken together, these findings extend the contribution of the study beyond a comparison of oversampling algorithms. The analysis shows that class-imbalance interventions can alter different dimensions of multiclass performance in different directions: gains in minority-class sensitivity may occur without corresponding improvement in overall agreement, precision, or specificity. Moreover, the repeated-cross-validation and corrected paired comparisons showed that the configuration with the highest cross-validated MCC was not uniformly superior to all strong alternatives. The contribution of the present work is therefore the characterization of these model-, class-, and metric-dependent trade-offs within a common validation framework, rather than the identification of a universally superior resampling strategy.

The pattern of errors also differed across hematological categories. In the selected unresampled CatBoost model, Class 0 showed the highest sensitivity and F1-score, whereas Class 1 showed the lowest values for these two metrics. In contrast, the Hb E carrier-related Class 2 had the lowest one-vs-rest AUC. The confusion matrix further showed that a substantial proportion of Class 1 observations were assigned to Class 2, while Class 2 observations were most frequently misclassified as Class 0. Therefore, no single class can be characterized as uniformly the most difficult across all evaluation measures. The lower discrimination observed for some of the non-normal categories may be related to overlap in CBC-derived hematological patterns. In particular, the Hb E carrier-related category may include individuals with relatively mild or overlapping hematological abnormalities [2], although the present data do not allow the biological basis of individual misclassifications to be established.

The complementary evaluation metrics also highlight why overall accuracy alone is insufficient for this task. Although the selected model achieved an accuracy of 0.7950, the class-specific sensitivities ranged from 0.4028 to 0.9767. At the same time, the micro-average ROC–AUC was 0.9554 and the macro-average ROC–AUC was 0.9227. These relatively high ROC–AUC values should therefore not be interpreted in isolation as indicating uniformly strong case detection across classes, because ROC–AUC is threshold independent whereas sensitivity depends on the operating decision rule. These differences reflect the fact that threshold-independent discrimination, overall classification agreement, and class-specific identification assess different aspects of model behavior. In an imbalanced multiclass setting, reporting MCC, Cohen’s kappa, class-specific sensitivity and F1-score, and ROC–AUC alongside accuracy therefore provides a more complete description of predictive performance. Similar concerns regarding class-sensitive evaluation have been noted in previous ML studies of thalassemia-related classification [13].

The class-specific results limit the extent to which the present model can be interpreted in a screening context. In the selected unresampled CatBoost model, sensitivity was 0.4028 for suspected α-thalassemia (Class 1), 0.6740 for the Hb E carrier-related Class 2, and 0.5000 for Class 3. Thus, although the model showed relatively high overall discrimination, its ability to identify individuals within these non-normal categories was substantially more limited, particularly for Classes 1 and 3. This is especially relevant for screening-oriented applications, in which false-negative classifications may have clinical consequences. Accordingly, the present findings should be interpreted as an evaluation of multiclass discrimination based on routine CBC-derived variables rather than as evidence of readiness for clinical screening or referral. Any future clinical use would require external validation, calibration, and prospective evaluation of decision thresholds.

The present study does not establish a clinically validated probability threshold or an acceptable false-negative rate. These quantities would require prospective evaluation in a clinically defined target population, with explicit consideration of the consequences of missed cases. Accordingly, the threshold analyses summarized in Supplementary Table S3 are interpreted as descriptive assessments of the trade-off between sensitivity and other performance measures rather than as recommendations for clinical deployment. Given the observed class-specific sensitivities, model outputs should not be interpreted as rule-out decisions. If such a framework were evaluated prospectively, its output would more appropriately serve as an adjunctive triage signal, while confirmatory hematological assessment, hemoglobin analysis, and molecular testing, where clinically indicated, would remain necessary. Accordingly, a model-generated non-normal probability would not constitute a diagnosis but could, after prospective validation of an appropriate operating threshold, be used to identify individuals for further hematological evaluation, whereas a low model probability should not by itself be used to exclude a hemoglobinopathy.

Several limitations should be considered when interpreting these findings. First, the primary analysis was based on a single publicly available source dataset, and the hold-out test set was derived from the same source population as the training data. Although the hold-out set was kept separate from preprocessing, resampling, hyperparameter optimization, and model selection, it originated from the same source population as the training data and therefore represents internal hold-out evaluation rather than external validation. Differences in patient characteristics, disease prevalence, referral patterns, laboratory analyzers, and diagnostic procedures across clinical settings may alter both the distribution of CBC variables and the performance of the resulting classifier. Accordingly, the primary results should not be interpreted as evidence of clinical applicability. Further external, multicenter, and prospectively collected validation would be required before clinical implementation could be considered. Recent biomedical ML studies have likewise shown that favorable internal performance may not translate into stable generalization under stricter validation procedures, further emphasizing the importance of rigorous validation design [60]. Second, the reference labels were inherited from the source dataset and were not independently reassessed in the present study. In addition, because CBC findings contributed to the operational definition of at least some of the original categories, particularly the distinction between Classes 0 and 1, a degree of incorporation between predictor information and reference labeling cannot be excluded and may influence the observed discrimination. Although hemoglobin-typing results were used for class assignment in the source cohort, definitive α-thalassemia status cannot be established from hemoglobin typing alone and requires molecular testing. Because individual-level information on molecular confirmation and diagnostic adjudication was not available in the public dataset, potential label uncertainty, particularly within categories involving possible or co-inherited α-thalassemia, could not be independently quantified. Third, the primary class-imbalance comparison was restricted to an unresampled baseline and four synthetic oversampling strategies. Additional sensitivity analyses were included to examine SMOTENC, class-weighted learning, and the potential contribution of biological sex; however, other approaches, including undersampling and hybrid resampling methods, were not systematically evaluated. Finally, the interpretability analyses were performed post hoc and were intended to characterize model behavior rather than establish biological or causal relationships. The resulting feature-attribution patterns should therefore be interpreted cautiously, and their reproducibility should be examined in independent cohorts.

Future work should evaluate the framework in externally collected and, where possible, multicenter cohorts representing different prevalence settings, demographic characteristics, and hematology analyzer platforms. Broader comparisons of class-imbalance strategies, including additional cost-sensitive, undersampling, and hybrid approaches, may also be informative. Prospective studies would be required to determine whether probability calibration and decision thresholds remain stable in clinically defined populations and to establish acceptable trade-offs between sensitivity and false-negative rates. The reproducibility of the feature-attribution patterns identified in the present analysis should likewise be examined in independent datasets before drawing broader conclusions about their hematological relevance.

## 5. Conclusions

This study compared seven supervised ML algorithms under an unresampled baseline and four synthetic oversampling strategies for the multiclass classification of four dataset-defined hematological profiles: normal hematological profiles, suspected α-thalassemia profiles, Hb E carrier-related profiles, and other thalassemia-related/hemoglobinopathic profiles. Based on the predefined cross-validated MCC criterion, CatBoost without resampling was selected as the final configuration. Synthetic oversampling did not provide a consistent increase in overall CV MCC, although some strategies were associated with higher sensitivity for specific minority classes. These findings indicate that the effect of class-imbalance handling is better characterized as a model-, class-, and metric-dependent trade-off than as a uniform improvement in predictive performance.

Class-specific performance varied across the four categories and according to the metric considered. In the selected model, sensitivity was 0.4028 for suspected α-thalassemia, 0.6740 for the Hb E carrier-related category, and 0.5000 for the other thalassemia-related/hemoglobinopathic category; suspected α-thalassemia also showed the lowest F1-score, whereas the Hb E carrier-related class showed the lowest one-vs-rest AUC. These findings indicate that class imbalance and overlapping hematological patterns remain relevant challenges in multiclass CBC-based classification.

The findings indicate that routine CBC-derived variables contain information relevant to multiclass discrimination among the hematological categories considered; however, the relatively low sensitivity observed for some minority classes limits conclusions regarding screening or referral use. External validation, assessment of calibration stability across independent clinical settings, and prospective evaluation of clinically relevant decision thresholds would be required before considering such an application. Further studies should also include multicenter and prospectively assembled cohorts and may examine cost-sensitive learning and explainability methods to better characterize model behavior and its potential clinical relevance.

## Figures and Tables

**Figure 1:**
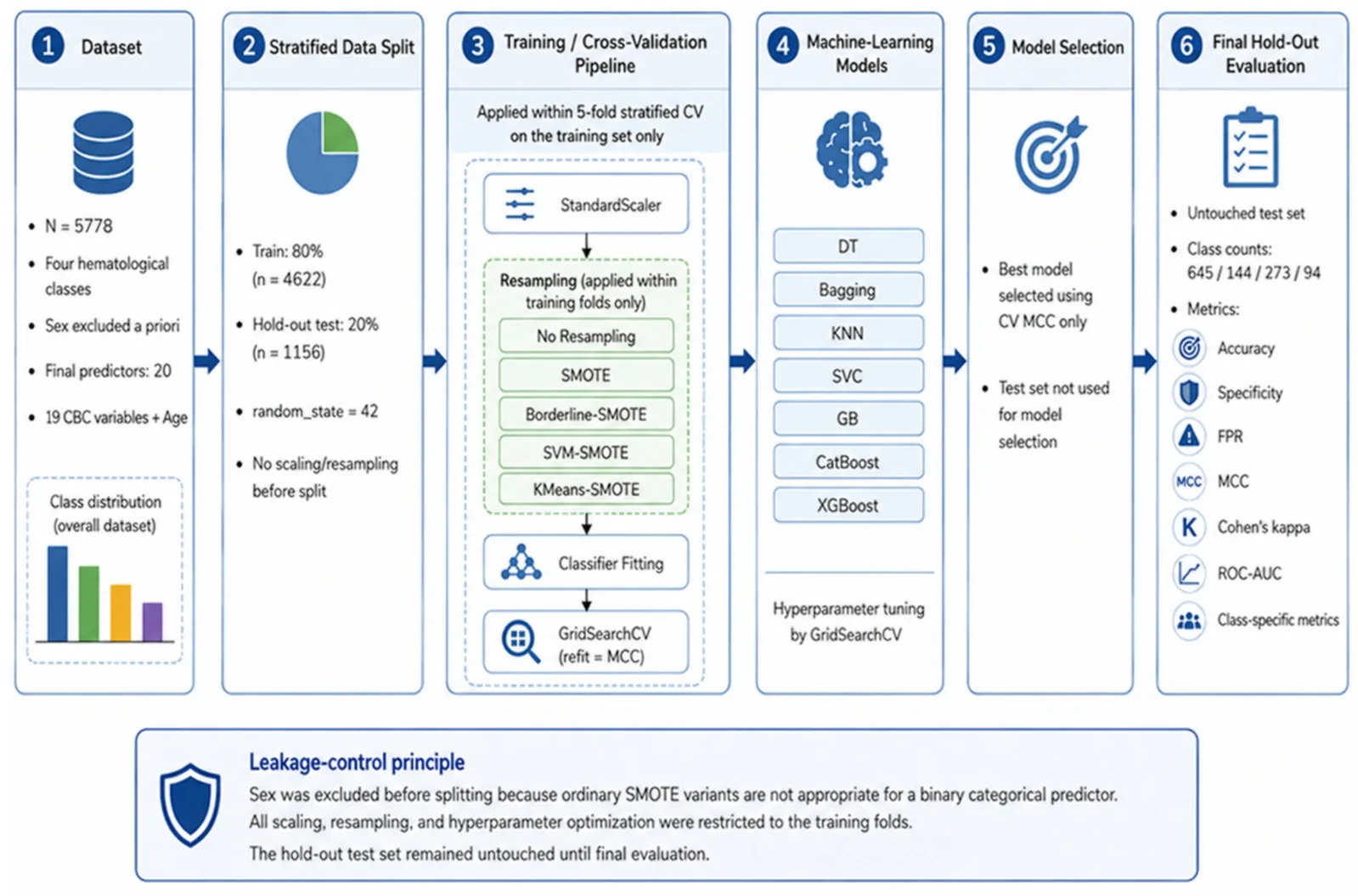
Leakage-free workflow of the machine learning framework for multiclass classification of hematological profiles using routine CBC-derived parameters and age.

**Figure 2:**
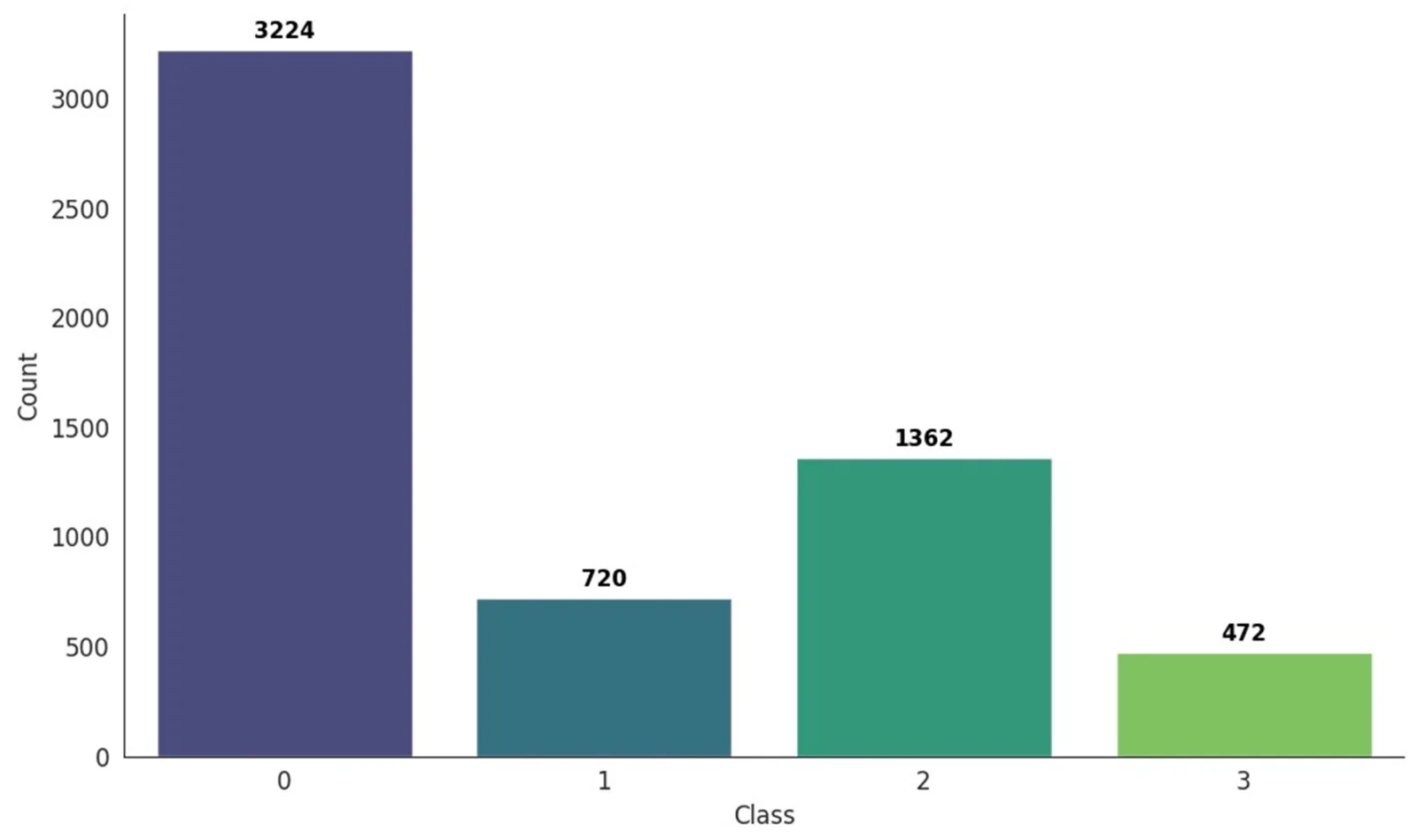
Distribution of observations across the four hematological classes.

**Figure 3:**
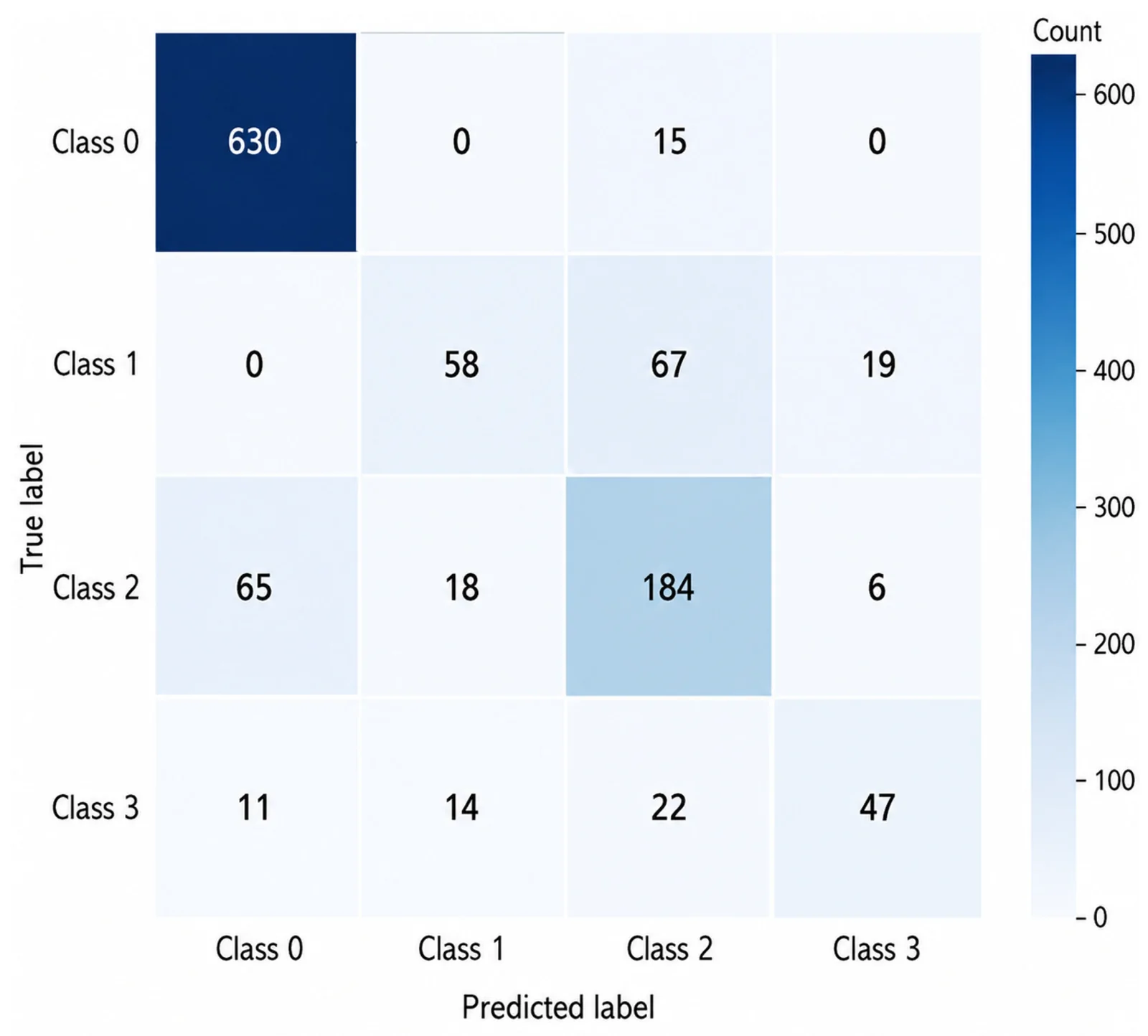
Confusion matrix of the selected CatBoost model without resampling on the untouched internal hold-out test set.

**Figure 4:**
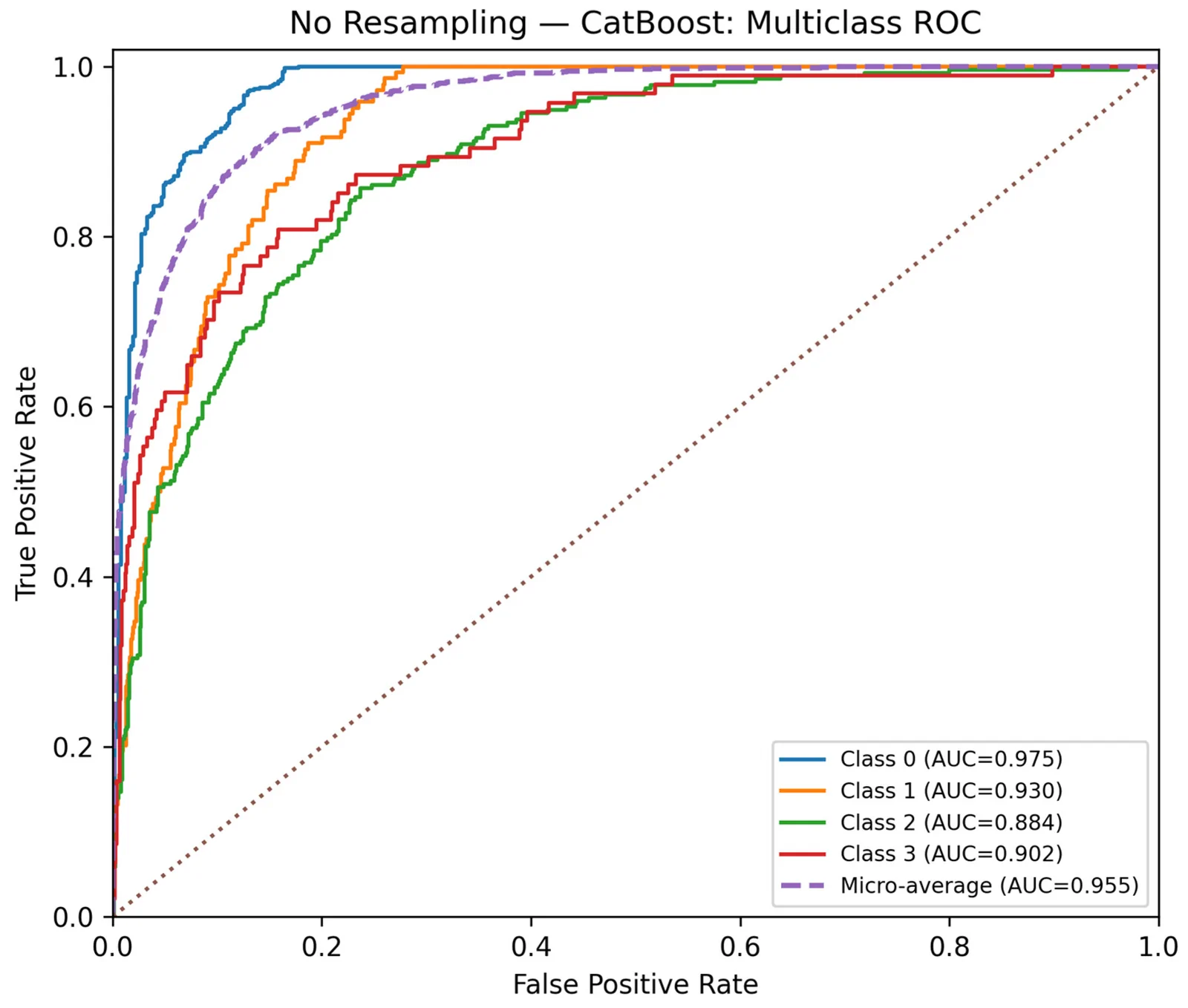
Class-specific and micro-average one-vs-rest ROC curves of the selected CatBoost model without resampling on the untouched internal hold-out test set. The dotted diagonal line represents the no-discrimination reference.

**Table 1:** Summary of retained input features and clinical relevance.

Feature	Full Name	Description/Clinical Meaning
RBC	Red Blood Cell count	Red blood cell count; a key parameter in screening for anemia and thalassemia
HGB	Hemoglobin	Hemoglobin concentration in the blood
HCT	Hematocrit	Ratio of red blood cells to total blood volume
MCV	Mean Corpuscular Volume	Mean corpuscular volume
MCH	Mean Corpuscular Hemoglobin	Hemoglobin content per red blood cell
MCHC	Mean Corpuscular Hemoglobin Concentration	Intracellular hemoglobin concentration
RDW	Red Cell Distribution Width	Red blood cell size distribution
PLT	Platelet count	Platelet count
WBC	White Blood Cell count	Total white blood cell count
NEU	Neutrophil (%)	Percentage of neutrophils
abs NEU	Absolute neutrophil count	Absolute neutrophil count
LYMP	Lymphocyte (%)	Percentage of lymphocytes
abs LYMP	Absolute lymphocyte count	Absolute lymphocyte count
MONO	Monocyte (%)	Percentage of monocytes
abs MONO	Absolute monocyte count	Absolute monocyte count
EOS	Eosinophil (%)	Percentage of eosinophils
abs EOS	Absolute eosinophil count	Absolute eosinophil count
BASO	Basophil (%)	Percentage of basophils
abs BASO	Absolute basophil count	Absolute basophil count
Age	Age (years)	Continuous age variable retained as a clinical covariate

**Table 2:** Hyperparameter search spaces used in GridSearchCV.

Model	Hyperparameter Search Space
GB	learning_rate: [0.05, 0.1]; n_estimators: [100, 200]
DT	criterion: [gini, entropy, log_loss]; max_depth: [None, 5, 10, 20]; min_samples_split: [2, 5, 10]; min_samples_leaf: [1, 2, 4]
Bagging	n_estimators: [50, 100, 200]; max_samples: [0.8, 1.0]; max_features: [0.8, 1.0]
KNN	n_neighbors: [3, 5, 7, 9]; p: [1, 2]; weights: [uniform, distance]
SVC	kernel: [rbf]; C: [1, 10, 100]; gamma: [0.01, 0.1, 1]
CatBoost	iterations: [200, 400]; learning_rate: [0.05, 0.1]; depth: [6, 8]; l2_leaf_reg: [1, 3]
XGBoost	n_estimators: [200, 400]; max_depth: [5, 7, 9]; learning_rate: [0.05, 0.1]; subsample: [0.8, 1.0]; colsample_bytree: [0.8, 1.0]

**Table 3:** Cross-validated MCC values of the seven classifiers under the evaluated class-imbalance strategies.

Classifier	No Resampling	SMOTE	Borderline-SMOTE	SVM-SMOTE	KMeans-SMOTE	Mean Across Strategies
DT	0.6145	0.5683	0.5569	0.5853	0.5940	0.5838
Bagging	0.6267	0.6027	0.6042	0.6179	0.6144	0.6132
KNN	0.5576	0.4592	0.4676	0.4985	0.5489	0.5064
SVC	0.6268	0.5955	0.5790	0.6139	0.6009	0.6032
GB	0.6336	0.6029	0.6026	0.6217	0.6299	0.6181
CatBoost	**0.6378**	**0.6159**	**0.6122**	**0.6293**	**0.6311**	**0.6252**
XGBoost	0.6313	0.6133	0.6095	0.6272	0.6287	0.6220
Mean across classifiers	0.6183	0.5797	0.5760	0.5991	0.6069	—

Note: Values represent mean MCC obtained from five-fold stratified cross-validation on the training set. Bold values in the classifier rows indicate the highest CV MCC within each class-imbalance strategy. The final row represents the mean CV MCC across the seven classifiers for each strategy. Hold-out test results were not used for classifier or strategy selection.

**Table 4:** Internal hold-out test performance of the CatBoost model selected under each class-imbalance strategy based on cross-validated MCC.

Strategy	CV MCC	Test Accuracy	Macro Specificity	Macro FPR	Test MCC	Cohen’s κ	Micro-AUC	Macro-AUC
No Resampling	0.6378	0.7950	0.9196	0.0804	0.6537	0.6499	0.9554	0.9227
SMOTE	0.6159	0.7777	0.9220	0.0780	0.6369	0.6364	0.9471	0.9129
Borderline-SMOTE	0.6122	0.7811	0.9216	0.0784	0.6388	0.6386	0.9493	0.9117
SVM-SMOTE	0.6293	0.7881	0.9224	0.0776	0.6479	0.6466	0.9508	0.9154
KMeans-SMOTE	0.6311	0.7846	0.9190	0.0810	0.6402	0.6377	0.9498	0.9119

Note: The model within each strategy was selected according to the highest CV MCC obtained on the training data. Specificity and FPR are macro-averaged across the four classes using a one-vs-rest formulation. Micro- and macro-AUC values were calculated from one-vs-rest class probabilities on the untouched internal hold-out test set. Hold-out test metrics were not used for model or strategy selection.

**Table 5:** Class-specific internal hold-out test performance of CatBoost under the evaluated class-imbalance strategies.

Strategy	Class	Sensitivity	Specificity	FPR	Precision	F1-Score	AUC
No Resampling	Class 0	0.9767	0.8513	0.1487	0.8924	0.9326	0.9752
	Class 1	0.4028	0.9684	0.0316	0.6444	0.4957	0.9297
	Class 2	0.6740	0.8822	0.1178	0.6389	0.6560	0.8839
	Class 3	0.5000	0.9765	0.0235	0.6528	0.5663	0.9018
SMOTE	Class 0	0.9318	0.9002	0.0998	0.9218	0.9268	0.9737
	Class 1	0.5556	0.9427	0.0573	0.5797	0.5674	0.9208
	Class 2	0.6081	0.9071	0.0929	0.6694	0.6372	0.8837
	Class 3	0.5532	0.9379	0.0621	0.4407	0.4906	0.8734
Borderline-SMOTE	Class 0	0.9426	0.8943	0.1057	0.9184	0.9304	0.9748
	Class 1	0.5069	0.9437	0.0563	0.5615	0.5328	0.9115
	Class 2	0.6227	0.8935	0.1065	0.6439	0.6331	0.8745
	Class 3	0.5532	0.9548	0.0452	0.5200	0.5361	0.8860
SVM-SMOTE	Class 0	0.9457	0.8924	0.1076	0.9173	0.9313	0.9747
	Class 1	0.4514	0.9555	0.0445	0.5909	0.5118	0.9142
	Class 2	0.6960	0.8720	0.1280	0.6271	0.6597	0.8803
	Class 3	0.4894	0.9699	0.0301	0.5897	0.5349	0.8922
KMeans-SMOTE	Class 0	0.9488	0.8767	0.1233	0.9067	0.9273	0.9743
	Class 1	0.4097	0.9625	0.0375	0.6082	0.4896	0.9141
	Class 2	0.6960	0.8584	0.1416	0.6032	0.6463	0.8735
	Class 3	0.4894	0.9783	0.0217	0.6667	0.5644	0.8858

Note: Class 0, normal hematological profile; Class 1, suspected α-thalassemia; Class 2, Hb E carrier-related profile; Class 3, other thalassemia-related/hemoglobinopathic profile. Sensitivity, specificity, FPR, precision, and F1-score were calculated using a one-vs-rest formulation. AUC represents class-specific one-vs-rest ROC–AUC on the untouched internal hold-out test set.

## Data Availability

We used publicly available dataset called the “CBC indices data set”. The dataset is available at https://data.mendeley.com/datasets/p85w7tbjbh/1, (accessed on 18 September 2026), DOI: 10.17632/p85w7tbjbh.1 [33].

## Supplementary Materials

The following supporting information can be downloaded at https://www.mdpi.com/article/10.3390/bioengineering13101118/s1: Figure S1: Univariate distributions of the 20 predictors included in the final analysis; Table S1: Corrected repeated cross-validation paired comparisons of MCC between the selected No Resampling–CatBoost configuration and all alternative model–resampling configurations; Table S1A: Repeated cross-validation stability of MCC across all 35 classifier–strategy configurations; Table S2: Sensitivity analyses of predictor composition and alternative class-imbalance handling strategies; Figure S2: Class-specific one-vs-rest calibration curves for the selected No Resampling–CatBoost model on the untouched internal hold-out set; Table S3: Exploratory one-vs-rest probability-threshold trade-offs for the less frequent hematological classes on the untouched internal hold-out set; Figure S3: Global SHAP feature importance for the selected No Resampling–CatBoost model on the untouched internal hold-out set; Figure S4: Model-agnostic permutation importance for the selected No Resampling–CatBoost model on the untouched internal hold-out set; Table S4: Five highest class-specific mean absolute SHAP values for the selected No Resampling–CatBoost model on the untouched internal hold-out set; Figure S5: Class-specific and micro-average one-vs-rest precision–recall curves for the selected No Resampling–CatBoost model on the untouched internal hold-out set.
