# Supplementary material for: Machine Learning-Based Multiclass Classification of Hb E Carrier Status and Thalassemia-Related Hematological Profiles Using Routine Complete Blood Count Parameters
Source: Bioengineering (Basel). 2026 Sep 24;13(10):1118. doi: 10.3390/bioengineering13101118 (PMC13701359; doi:10.3390/bioengineering13101118)
Supplement: Supplementary file 1 [file bioengineering-13-01118-s001.zip › bioengineering-4563180-supplementary.pdf]

Figure S1. Univariate distributions of the 20 predictors included in the final analysis. Histograms with kernel density estimates are shown for 19 routine CBC-derived hematological parameters and age.

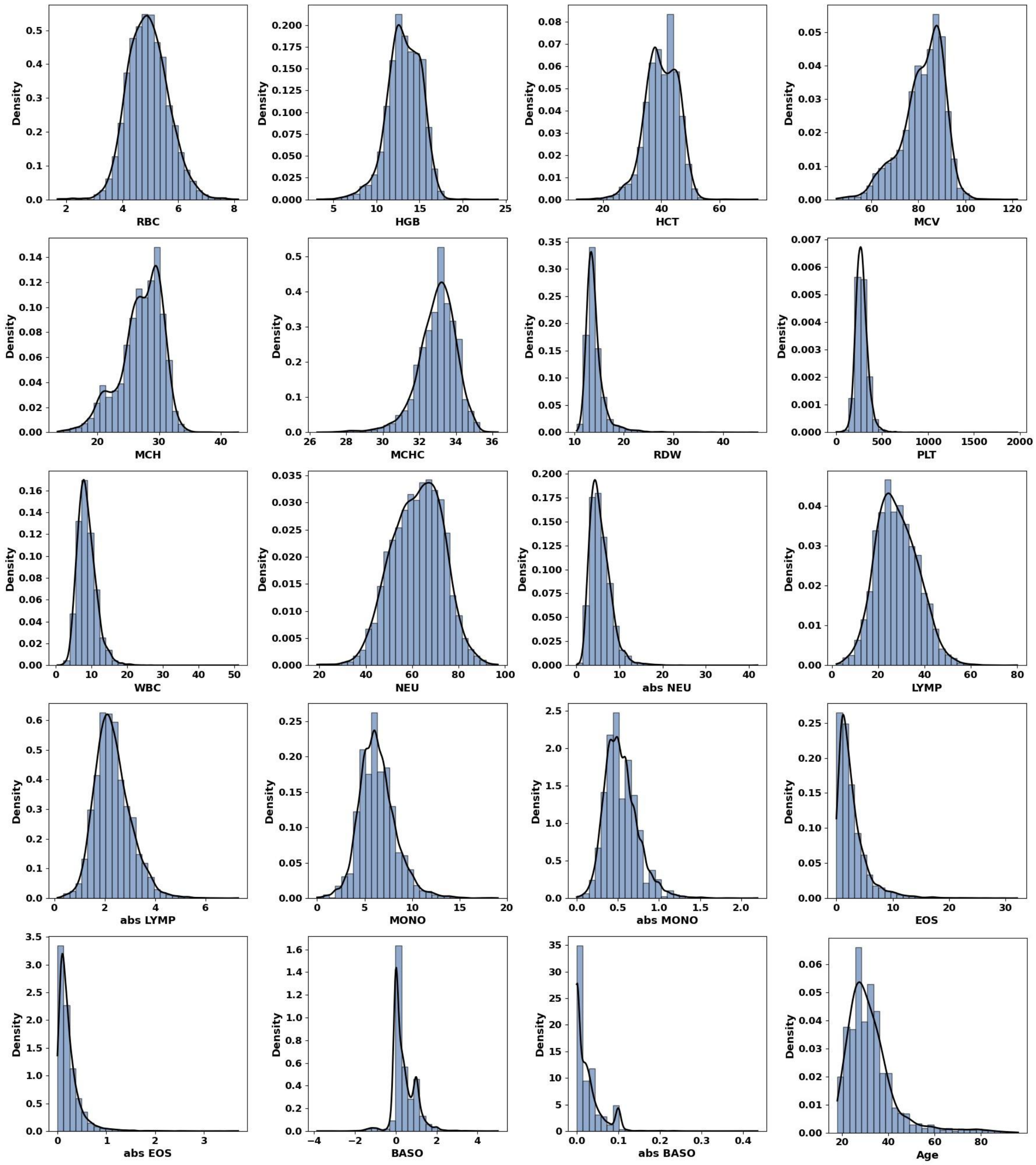

Supplementary Table S1. Corrected repeated cross-validation paired comparisons of MCC between the selected No Resampling–CatBoost configuration and all alternative model–resampling configurations.

Comparator	Mean MCC difference	Corrected t	Raw p	Holm-adjusted p
SMOTE – KNN	0.1835	16.8585	<0.001	<0.001
Borderline-SMOTE – KNN	0.1742	13.7622	<0.001	<0.001
SVM-SMOTE – KNN	0.1436	11.8754	<0.001	<0.001
KMeans-SMOTE – KNN	0.0974	9.2721	<0.001	<0.001
No Resampling – KNN	0.0764	7.6449	<0.001	<0.001
SMOTE – DT	0.0908	7.2331	<0.001	<0.001
Borderline-SMOTE – SVC	0.0617	6.4908	<0.001	<0.001
SVM-SMOTE – DT	0.0513	5.3355	<0.001	<0.001
Borderline-SMOTE – DT	0.0804	4.8702	<0.001	<0.001
Borderline-SMOTE – GB	0.0476	4.8506	<0.001	<0.001
SMOTE – SVC	0.0466	4.7261	<0.001	<0.001
KMeans-SMOTE – SVC	0.0362	4.5639	<0.001	<0.001
SMOTE – Bagging	0.0415	4.4473	<0.001	0.0011
SMOTE – GB	0.0422	4.4052	<0.001	0.0012
KMeans-SMOTE – DT	0.0449	4.4022	<0.001	0.0012
SMOTE – CatBoost	0.0363	4.1411	<0.001	0.0026
Borderline-SMOTE – CatBoost	0.0382	4.0480	<0.001	0.0033
Borderline-SMOTE – Bagging	0.0379	3.7934	<0.001	0.0070
Borderline-SMOTE – XGB	0.0355	3.7061	<0.001	0.0086
SMOTE – XGB	0.0335	3.5791	<0.001	0.0118
SVM-SMOTE – Bagging	0.0294	3.5693	<0.001	0.0118
SVM-SMOTE – SVC	0.0298	3.4178	0.0013	0.0166
KMeans-SMOTE – Bagging	0.0217	3.2032	0.0024	0.0287
No Resampling – DT	0.0240	3.1400	0.0029	0.0315
SVM-SMOTE – XGB	0.0262	2.7979	0.0073	0.0733
SVM-SMOTE – GB	0.0217	2.6951	0.0096	0.0865
KMeans-SMOTE – GB	0.0184	2.5559	0.0137	0.1100
No Resampling – SVC	0.0187	2.4586	0.0175	0.1227
KMeans-SMOTE – XGB	0.0148	1.9450	0.0575	0.2673
SVM-SMOTE – CatBoost	0.0177	2.0591	0.0448	0.2673
KMeans-SMOTE – CatBoost	0.0154	2.0619	0.0445	0.2673
No Resampling – Bagging	0.0144	1.6994	0.0956	0.2868
No Resampling – GB	0.0063	1.1237	0.2666	0.5332
No Resampling – XGB	0.0062	0.9280	0.3579	0.5332

Note: The selected configuration was No Resampling–CatBoost. Mean MCC differences were calculated as the MCC of the selected configuration minus that of the comparator; therefore, positive values indicate higher mean MCC for the selected configuration. Comparisons were based on repeated stratified 5-fold cross-validation with 10 repetitions. Corrected t statistics were obtained using the corrected resampled t-test. P-values were adjusted for multiple comparisons using the Holm procedure. Values of $p < 0.001$ are reported as <0.001.

Supplementary Table S1A. Repeated cross-validation stability of MCC across all 35 classifier–strategy configurations.

Strategy	Model	Mean MCC	SD	Corrected 95% CI
No Resampling	CatBoost	0.6380	0.0174	0.6197–0.6562
No Resampling	XGB	0.6318	0.0182	0.6128–0.6507
No Resampling	GB	0.6316	0.0167	0.6142–0.6490
No Resampling	Bagging	0.6236	0.0179	0.6049–0.6423
KMeans-SMOTE	XGB	0.6232	0.0166	0.6059–0.6405
KMeans-SMOTE	CatBoost	0.6225	0.0181	0.6036–0.6415
SVM-SMOTE	CatBoost	0.6203	0.0182	0.6012–0.6393
KMeans-SMOTE	GB	0.6196	0.0183	0.6004–0.6387
No Resampling	SVC	0.6192	0.0160	0.6025–0.6360
KMeans-SMOTE	Bagging	0.6163	0.0167	0.5988–0.6337
SVM-SMOTE	GB	0.6162	0.0196	0.5958–0.6367
No Resampling	DT	0.6139	0.0193	0.5937–0.6341
SVM-SMOTE	XGB	0.6118	0.0196	0.5913–0.6323
SVM-SMOTE	Bagging	0.6085	0.0159	0.5919–0.6252
SVM-SMOTE	SVC	0.6082	0.0205	0.5868–0.6296
SMOTE	XGB	0.6044	0.0199	0.5836–0.6252
Borderline-SMOTE	XGB	0.6024	0.0174	0.5843–0.6206
KMeans-SMOTE	SVC	0.6017	0.0187	0.5822–0.6212
SMOTE	CatBoost	0.6016	0.0184	0.5824–0.6209
Borderline-SMOTE	Bagging	0.6001	0.0178	0.5815–0.6187
Borderline-SMOTE	CatBoost	0.5998	0.0186	0.5803–0.6192
SMOTE	Bagging	0.5965	0.0142	0.5816–0.6114
SMOTE	GB	0.5957	0.0169	0.5781–0.6134
KMeans-SMOTE	DT	0.5930	0.0195	0.5726–0.6134
SMOTE	SVC	0.5914	0.0197	0.5708–0.6120
Borderline-SMOTE	GB	0.5903	0.0197	0.5698–0.6109
SVM-SMOTE	DT	0.5867	0.0198	0.5660–0.6074
Borderline-SMOTE	SVC	0.5762	0.0185	0.5569–0.5956
No Resampling	KNN	0.5616	0.0183	0.5425–0.5807
Borderline-SMOTE	DT	0.5576	0.0274	0.5290–0.5862
SMOTE	DT	0.5472	0.0267	0.5194–0.5750
KMeans-SMOTE	KNN	0.5405	0.0212	0.5184–0.5627
SVM-SMOTE	KNN	0.4944	0.0193	0.4742–0.5146
Borderline-SMOTE	KNN	0.4638	0.0176	0.4454–0.4822
SMOTE	KNN	0.4545	0.0195	0.4342–0.4748

Note: Values were obtained from repeated stratified 5-fold cross-validation with 10 repetitions using the tuned hyperparameters from the initial model-selection stage. Mean MCC and standard deviation (SD) summarize the 50 repeated-CV fold estimates for each configuration. Corrected 95% confidence intervals account for dependence among repeated cross-validation estimates and are intended as stability intervals rather than external-validation confidence intervals. Configurations are ordered by mean MCC; the selected No Resampling–CatBoost configuration is shown in bold.

Supplementary Table S2. Sensitivity analyses of predictor composition and alternative class-imbalance handling strategies

Configuration	Accuracy	Balanced Accuracy	MCC	Macro-F1	Class 0 Sensitivity	Class 1 Sensitivity	Class 2 Sensitivity	Class 3 Sensitivity
CBC only	0.7958	0.6315	0.6548	0.6571	0.9814	0.4236	0.6740	0.4468
CBC + Age (Primary)	0.7950	0.6384	0.6537	0.6627	0.9767	0.4028	0.6740	0.5000
CBC + Age + Sex	0.7933	0.6337	0.6504	0.6611	0.9767	0.4375	0.6630	0.4574
CBC + Age, Class-weighted CatBoost	0.7829	0.6468	0.6399	0.6565	0.9411	0.4653	0.6703	0.5106
CBC + Age + Sex, SMOTENC	0.7742	0.6497	0.6286	0.6468	0.9426	0.5417	0.5824	0.5319

Note: The primary configuration included 19 CBC-derived variables and Age. Sex inclusion, SMOTENC, and class-weighted CatBoost were evaluated as sensitivity analyses using the same internal training/hold-out split. Sensitivity denotes class-specific recall.

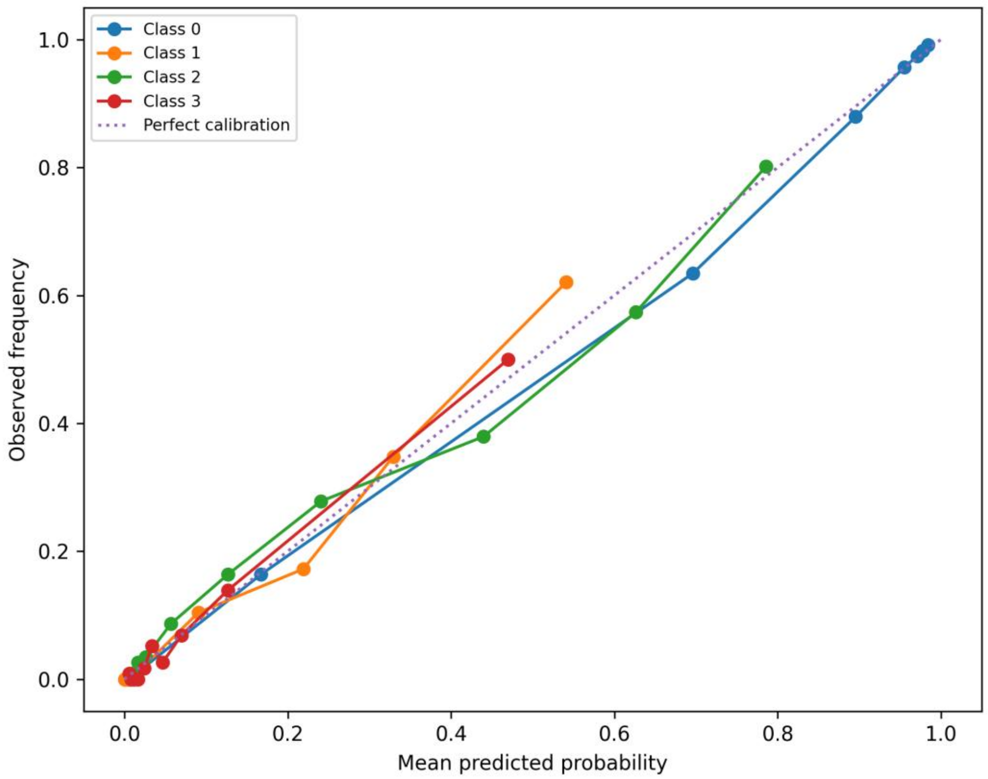

Figure S2. Class-specific one-vs-rest calibration curves for the selected No Resampling–CatBoost model on the untouched internal hold-out set. The diagonal reference line represents perfect agreement between mean predicted probability and observed class frequency. The curves characterize internal probability calibration and should not be interpreted as evidence of clinical calibration.

Supplementary Table S3. Exploratory one-vs-rest probability-threshold trade-offs for the less frequent hematological classes on the untouched internal hold-out set.

Class	Threshold	Sensitivity	Specificity	PPV	NPV	FPR	FNR
Class 1	0.20	0.8889	0.8202	0.4129	0.9811	0.1798	0.1111
Class 1	0.30	0.7292	0.9032	0.5172	0.9591	0.0968	0.2708
Class 1	0.40	0.4931	0.9585	0.6283	0.9300	0.0415	0.5069
Class 1	0.50	0.3056	0.9822	0.7097	0.9086	0.0178	0.6944
Class 2	0.20	0.8315	0.7735	0.5316	0.9369	0.2265	0.1685
Class 2	0.30	0.7546	0.8245	0.5706	0.9157	0.1755	0.2454
Class 2	0.40	0.6923	0.8652	0.6136	0.9009	0.1348	0.3077
Class 2	0.50	0.6374	0.8958	0.6541	0.8888	0.1042	0.3626
Class 3	0.20	0.6064	0.9501	0.5182	0.9646	0.0499	0.3936
Class 3	0.30	0.5638	0.9633	0.5761	0.9615	0.0367	0.4362
Class 3	0.40	0.5106	0.9783	0.6761	0.9576	0.0217	0.4894
Class 3	0.50	0.3723	0.9896	0.7609	0.9468	0.0104	0.6277

Note: Thresholds were evaluated descriptively and were not optimized or selected for clinical use. Sensitivity, specificity, positive predictive value (PPV), negative predictive value (NPV), false-positive rate (FPR), and false-negative rate (FNR) were calculated using one-vs-rest class probabilities. No clinically acceptable false-negative rate or operating threshold was prespecified.

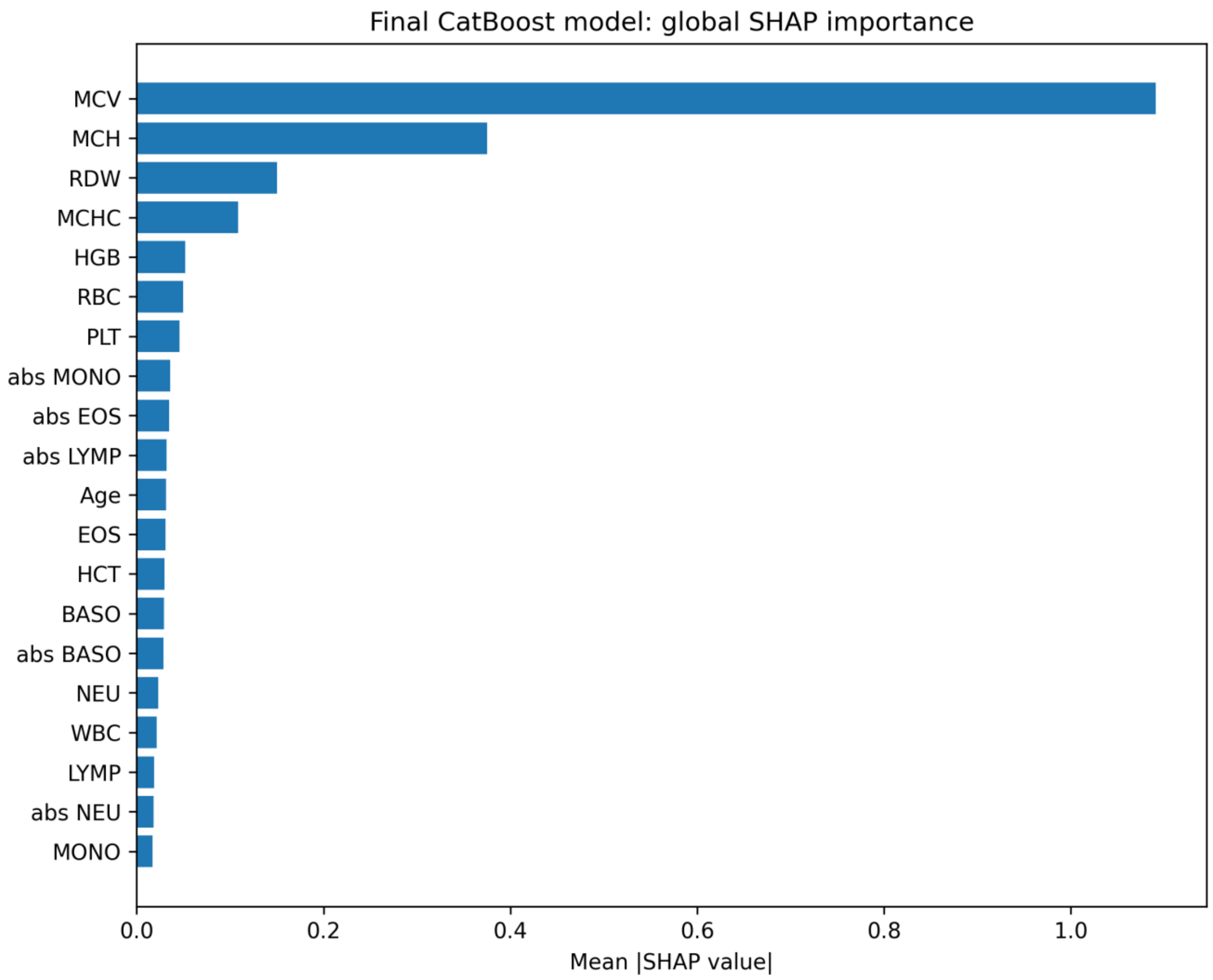

Figure S3. Global SHAP feature importance for the selected No Resampling–CatBoost model on the untouched internal hold-out set. Importance is summarized as the mean absolute SHAP value across the four dataset-defined hematological classes. Larger values indicate greater contribution to the model output; the values describe model behavior and should not be interpreted as causal effects.

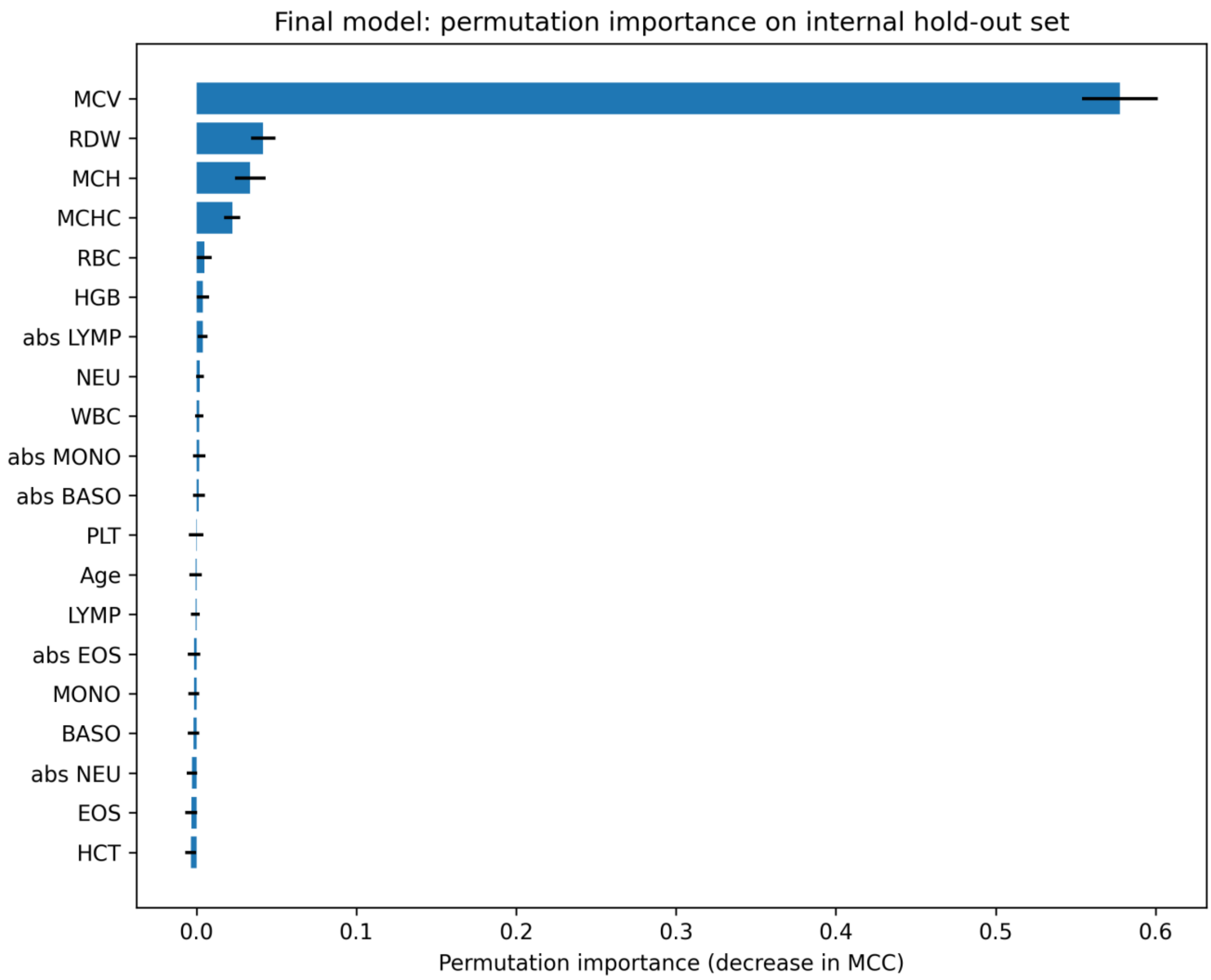

Figure S4. Model-agnostic permutation importance for the selected No Resampling–CatBoost model on the untouched internal hold-out set. Importance was quantified as the mean decrease in Matthews correlation coefficient (MCC) after permuting each predictor; error bars represent the standard deviation across 30 permutation repeats. Negative or near-zero values indicate little measurable contribution under this permutation-based assessment.

Supplementary Table S4. Five highest class-specific mean absolute SHAP values for the selected No Resampling–CatBoost model on the untouched internal hold-out set.

**Class**	**Rank**	**Feature**	**Mean ǀSHAPǀ**
Class 0	1	MCV	2.1290
Class 0	2	MCH	0.7128
Class 0	3	MCHC	0.1264
Class 0	4	RDW	0.1036
Class 0	5	HGB	0.0990
Class 1	1	MCV	1.4725
Class 1	2	MCH	0.3962
Class 1	3	RDW	0.1178
Class 1	4	RBC	0.0790
Class 1	5	MCHC	0.0771
Class 2	1	MCV	0.5424
Class 2	2	MCH	0.2329
Class 2	3	RDW	0.1755
Class 2	4	MCHC	0.0869
Class 2	5	BASO	0.0508
Class 3	1	MCV	0.2190
Class 3	2	RDW	0.2048
Class 3	3	MCH	0.1584
Class 3	4	MCHC	0.1443
Class 3	5	PLT	0.0777

Note: SHAP values were calculated after model selection and summarize the magnitude of feature contributions to the fitted CatBoost model. They do not establish biological causality. The class labels correspond to the dataset-defined hematological categories used in the primary analysis.

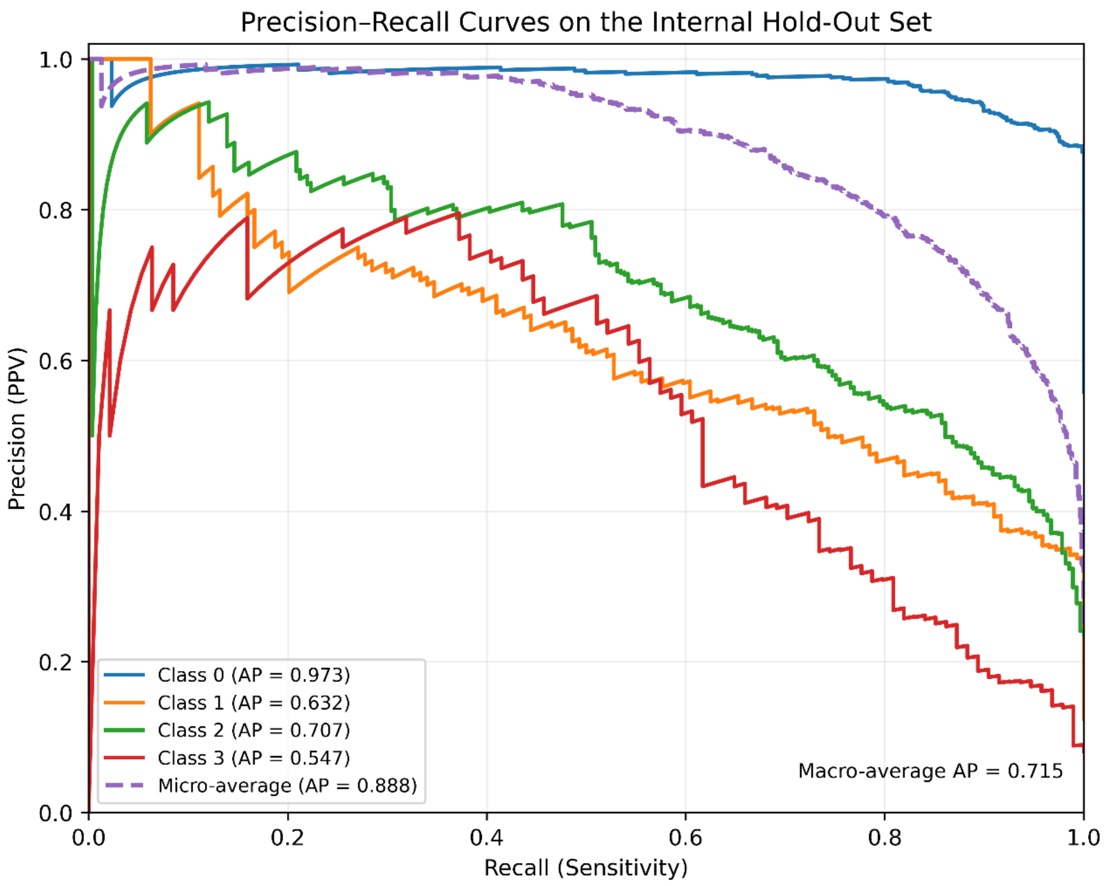

Figure S5. Class-specific and micro-average one-vs-rest precision–recall curves for the selected No Resampling–CatBoost model on the untouched internal hold-out set. Average precision (AP) was 0.9728 for Class 0, 0.6319 for Class 1, 0.7071 for Class 2, and 0.5467 for Class 3; the micro- and macro-average AP values were 0.8876 and 0.7146, respectively.
